# Intravenous immunoglobulin as first-line acute treatment in adults with autoimmune encephalitis caused by antibodies to NMDAR, LGI1 and CASPR2

**DOI:** 10.1007/s00415-025-13032-0

**Published:** 2025-03-25

**Authors:** Joyce Christin Rittel, Dominica Hudasch, Kathrin Doppler, Florian Then Bergh, Martin Lesser, Orhan Aktas, Michael Nagel, Hagen B. Huttner, Kevin Rostasy, Simone Tauber, Manuel A. Friese, Michael Malter, Marie Madlener, Andrea Kraft, Frank Hoffmann, Jan Lewerenz, Makbule Senel, Jonathan Wickel, Christian Geis, Andreas Moser, Klaus-Peter Wandinger, Thorsten Bartsch, Frank Leypoldt, Franziska Thaler, Tania Kümpfel, Sven Meuth, Nico Melzer, Carsten Finke, Harald Prüss, Martin Stangel, Kurt-Wolfram Sühs, German Network for Research on Autoimmune Encephalitis (GENERATE)

**Affiliations:** 1 Department of Neurology, Hannover Medical School, Hannover, Germany; 2 Department of Neurology, University Hospital of Würzburg, Würzburg, Germany; 3 Department of Neurology, University of Leipzig, Leipzig, Germany; 4 Department of Neurology, University Hospital Carl Gustav Carus, Technische Universität Dresden, Dresden, Germany; 5 Department of Neurology, Medical Faculty and University Hospital, Heinrich-Heine-Universität Düsseldorf, 40225 Düsseldorf, Germany; 6 Department of Neurology, Hospital Osnabrück, Osnabrück, Germany; 7 Department of Neurology, University of Erlangen-Nuremberg, Erlangen, Germany; 8 Department of Neurology, Justus Liebig University Giessen, Giessen, Germany; 9 Pediatric Neurology, Children’s Hospital Datteln, University Witten/Herdecke, Witten, Germany; 10 Department of Neurology, RWTH University Hospital, Aachen, Germany; 11 Institute of Neuroimmunology and Multiple Sclerosis, Center for Molecular Neurobiology Hamburg, University Medical Center Hamburg-Eppendorf, Hamburg, Germany; 12 Department of Neurology, Faculty of Medicine, University of Cologne and University Hospital of Cologne, Cologne, Germany; 13 Department of Neurology, Martha-Maria Hospital Halle-Dölau, Halle-Dölau, Germany; 14 Department of Neurology, University of Ulm, Ulm, Germany; 15 Section of Translational Neuroimmunology, Department of Neurology, Jena University Hospital, Jena, Germany; 16 Department of Neurology, University of Lübeck and University Medical Center of Schleswig-Holstein, Campus Lübeck, Lübeck, Germany; 17 Department of Neurology, Kiel University, Kiel, Germany; 18 Institute of Clinical Chemistry, University Hospital Schleswig-Holstein, Schleswig-Holstein, Germany; 19 Institute of Clinical Neuroimmunology, University Hospital, Ludwig-Maximilians-Universität Munich, Munich, Germany; 20 Biomedical Center (BMC), Medical Faculty, Ludwig-Maximilians-Universität Munich, Martinsried, Germany; 21 Department of Neurology with Institute of Translational Neurology, University Hospital, Muenster, Germany; 22 Department of Neurology and Experimental Neurology, Charité - Universitätsmedizin Berlin, Berlin, Germany; 23 German Center for Neurodegenerative Diseases (DZNE) Berlin, Berlin, Germany; 24 Translational Medicine, Novartis Institute of Biomedical Research, Basel, Switzerland

**Keywords:** Autoimmune encephalitis, NMDA, LGI-1, CASPR2, IVIG, Immunotherapy

## Abstract

**Background and objectives:**

Corticosteroids or plasma exchange are recommended for acute treatment of autoimmune encephalitis (AE). Intravenous immunoglobulins (IVIG) are suggested as an additional treatment option but data on treatment effect is scarce. The objective of the present study was to investigate the impact of the first-line treatment on the three most common forms of AE, in particular, to evaluate the effect of IVIG therapy in these diseases.

**Methods:**

A total of 1274 patients from the German Network for Autoimmune Encephalitis Research (GENERATE) were analyzed, and 388 patients were included in the study because they had either anti-NMDAR, anti-LGI1 or anti-CASPR2 antibodies and firs-line immunotherapy (ivMP monotherapy, ivMP + IVIG, ivMP + PE or ivMP + IVIG + PE) or no immunotherapy at all. For the statistical analyses, patients were stratified according to antibody type, distinguishing between anti-NMDAR (IgG1) and anti-LGI1 as well as anti-CASPR2 (predominantly IgG4). The primary endpoint was the clinical outcome at discharge, which was assessed using the modified Rankin Scale (mRS). The mRS scores were then compared between the different treatment groups over time, and the factors influencing the reduction in mRS at discharge were analyzed. Furthermore, a specific investigation was conducted to determine the differences in outcomes between patients treated with ivMP + IVIG and ivMP + PE, each split by antibody subtype.

**Results:**

In all treatment groups analyzed, significant improvements were observed at the time of discharge and after 12 months compared to disease onset, regardless of the type of first-line treatment. In untreated patients a significant improvement was not observed. The choice of IVIG or PE as an additional treatment to ivMP for anti-NMDAR encephalitis did not affect the primary outcome. In anti-LGI1 or anti-CASPR2 encephalitis, no influence on the primary outcome was observed when IVIG or PE was administered in addition to ivMP, too. However, a direct comparison of the individual antibody subgroups’ mRS reductions, depending on the treatment approach (ivMP + IVIG vs. ivMP + PE), revealed that a more significant mRS reduction was observed with ivMP + PE in anti-NMDAR encephalitis.

**Discussion:**

The retrospective data give evidence that there is no difference in outcome for the use of ivMP + PE over ivMP + IVIG or vice versa in the treatment of encephalitis caused by antibodies against NMDAR, LGI1 or CASPR2. Furthermore, the specific method of plasma exchange, whether plasmapheresis or immunoadsorption, did not affect the mRS at discharge.

**Supplementary Information:**

The online version contains supplementary material available at 10.1007/s00415-025-13032-0.

## Introduction

The majority of antibodies that have been identified as targeting cell surface-expressed molecules in the central nervous system (CNS) have been shown to be directly associated with the development of autoimmune encephalitis (AE) [1]. Along with the N-methyl-D-aspartate receptor (NMDAR), leucine-rich-glioma-inactivated-1 (LGI1) and contactin-associated-protein-like-2 (CASPR2) are the three most common target antigens in antibody-mediated encephalitis. The age of onset for anti-NMDAR encephalitis ranges from 0.6 to 85 years, for anti-LGI1 encephalitis from 30 to 80 years, and for anti-CASPR2 encephalitis from 46 to 77 years [2]. The clinical presentation of anti-NMDAR encephalitis is typically characterized by the subacute onset of psychotic symptoms, which may include hallucinations, paranoia or behavioural disturbances, frequently followed by additional neurological manifestations such as movement disorders or seizures. Patients with anti-LGI1 encephalitis often present with focal seizures, particularly of the faciobrachial dystonic type, in addition to complex focal seizures accompanied by cognitive or autonomic features. Anti-CASPR2 encephalitis features cognitive impairment, memory disorders, seizures, cerebellar symptoms and sleep disturbances [3].The autonomic nervous system as well as the peripheral nervous system can also be affected by antibodies against CASPR2. The treatment of autoimmune encephalitis is typically a multi-step process that aims to regulate the autoimmune response. In the absence of randomised controlled trials, current treatment protocols are largely based on clinical experience and retrospective analyses. First-line treatment typically comprises high-dose corticosteroids such as intravenous methylprednisolone (ivMP), in conjunction with plasmapheresis (PE) or intravenous immunoglobulin (IVIG) [4]. Corticosteroids are frequently the first choice due to their potent anti-inflammatory effects, while PE and IVIG are considered when rapid immunomodulation is necessary. In cases that are refractory to first-line treatment, second-line therapies such as rituximab and cyclophosphamide are employed. These agents aim to achieve more profound and prolonged suppression of the immune response, especially in cases that are severe or relapsing [5]. The response to therapy seems to vary depending on the type of antibodies involved. For instance, patients diagnosed with anti-LGI1 encephalitis frequently respond better to corticosteroids than to IVIG [6]. Intravenous immunoglobulin (IVIG) has become an essential component of acute exacerbation (AE) treatment due to its favourable administration profile and broad immunomodulatory effects. In contrast to plasmapheresis, IVIG does not necessitate central venous access, thereby rendering it a more pragmatic choice for patients exhibiting cognitive impairments, agitation, or compromised venous access [7, 8]. The mechanisms of IVIG action are diverse and include modulation of B and T cells, neutralization of autoantibodies, and inhibition of the complement system [9–11]. As demonstrated by Imbach et al. in 1980, IVIG was originally introduced for the treatment of immune thrombocytopenia [9]. Since then, it has been shown to be effective in a number of autoimmune and inflammatory diseases, including Guillain-Barré syndrome, chronic inflammatory demyelinating polyneuropathy (CIDP), multifocal motor neuropathy, dermatomyositis, and myasthenia gravis rapidly stabilizing the immune response and promote neurological recovery [12–15]. Observational data from NHS studies have shown that approximately 2% of AE patients received exclusive IVIG treatment, with cognitive and functional improvements documented in over 40% of these cases, despite limited outcome data availability. Furthermore, IVIG has been associated with reduced hospitalization durations and accelerated resumption of normal activities when administered concomitantly with corticosteroids. Nevertheless, the high cost of IVIG and its limited availability in certain healthcare systems represent challenges that must be addressed [16]. There is a lack of direct comparative data between IVIG and other treatment modalities, such as PE. This deficiency in robust evidence underscores the necessity for further research in this area. The present status of AE treatment, as evidenced by its empirical nature, asks for systematic clinical studies aimed at refining therapeutic strategies [4]. The following investigation seeks to define the most effective first-line therapies to ascertain whether IVIG confers superior or equivalent benefits in comparison to PE, particularly in specific subtypes of AE, in order to facilitate the precise tailoring of treatments by clinicians. The establishment of evidence-based guidelines would not only lead to improvements in patient outcomes but also ensure efficient resource utilization in the management of this complex and potentially life-threatening condition.

## Results

Demographic, clinical and laboratory characteristics: the patients were distributed between two groups according to their antibodies. The first group comprised 238 patients with anti-NMDAR encephalitis, while the second group consisted of 150 patients with anti-LGI1 or anti-CASPR2 encephalitis (anti-LGI1 107 patients, anti-CASPR2 43 patients). The demographic, clinical and CSF examination and their distribution among the treatments are presented in Table 1.

**Table 1:** Presentation of demographic, clinical and cerebrospinal fluid (CSF) data broken down by therapy group and by antibody

Anti-NMDAR	No therapy	ivMP	No therapy vs. ivMP	ivMP + IVIG	No therapy vs. ivMP + IVIG	ivMP + PE	No therapy vs. ivMP + PE	ivMP + PE + IVIG	No therapy vs. ivMP + PE + IVIG	Total
	*n* = 16	*n* = 31	*p*	*n* = 52	*p*	*n* = 82	*p*	*n* = 57	*p*	*n* = 222
Age at onset; years (mean)	46	38	0.1594	27	**0.0008**	34	**0.0156**	29	**0.0009**	31
Female; *n*; %	10; 61%	22; 71%	0.14	38; 73.1%	0.416	57; 69.5%	0.5811	45; 78.9%	0.1774	162; 73%
Clinical and CSF features										
Seizures; *n*; %	8; 44%	14; 45.2%	0.7527	27; 51.9%	0.8929	46; 56.1%	0.6538	46; 80.7%	**0.0134**	133; 59.9%
Psychiatric symptoms; *n*; %	9; 61%	23; 74.2%	0.2111	42; 80.8%	**0.0476**	67; 81.7%	**0.0256**	52; 91.2%	**0.0009**	184; 82.9%
Movement disorders; *n*; %	0	8; 25.8%	**0.0003**	17; 32.7%	**0.0071**	33; 40.2%	**0.001**	29; 50.9%	**< 0.0001**	87; 39.2%
Higher cognitive dysfunction *n*; %	8; 50%	17; 54.8%	0.3932	36; 69.2%	0.1592	58; 70.7%	0.1058	39; 68.4%	0.1739	150; 67.6%
Malignancy; *n*; %	1; 5%	2; 6.5%	1	8; 15.4%	0.6742	18; 22%	0.1854	14; 24.6%	0.1650	42; 18.9%
CSF elevated cell count; *n*; %	3; 28%	15; 48.4%	0.0624	34; 65.4%	**0.0014**	54; 65.9%	**0.0017**	41; 71.9%	**0.0003**	144; 64.9%
CSF elevated protein; *n*; %	5; 33%	21; 67.7%	0.0881	35; 67.3%	0.1474	61; 74.4%	**0.0008**	44; 77.2%	**0.0005**	161; 72.5%
CSF positive OCB; *n*; %	6; 33%	10; 32.3%	0.7193	26; 50%	0.3810	39; 47.6%	0.4601	28; 49.1%	0.4102	103; 36.4%

Anti-LGI1/CASPR2	No therapy	ivMP	No therapy vs. ivMP	ivMP + IVIG	No therapy vs. ivMP + IVIG	ivMP + PE	No therapy vs. ivMP + PE	ivMP + PE + IVIG	No therapy vs. ivMP + PE + IVIG	Total
	*n* = 14	*n* = 44		*n* = 31		*n* = 46		*n* = 15		*n* = 136
Age at onset; years (mean)	67	63	0.3894	62	0.5830	66	0.8665	56	**0.0162**	63
Female; *n*; %	3; 21%	14; 31.8%	0.5232	11; 35.5%	0.4921	21; 45.7%	0.1296	8; 53.3%	0.1281	54; 39.7%
Clinical and CSF features										
Seizures; *n*; %	11; 79%	27; 61.4%	0.3381	21; 67.7%	0.7236	28; 60.9%	0.3396	10; 66.7%	0.6817	86; 63.2%
Psychiatric symptoms; *n*; %	6; 43%	12; 27.3%	0.1953	20; 64.5%	0.1732	18; 39.1%	0.8032	10; 66.7%	0.2723	60; 44.1%
Movement disorders; *n*; %	3; 21%	6; 13.6%	0.6725	4; 12.9%	0.6593	6; 13%	0.6725	1; 6.7%	0.3295	17; 12.5%
Higher cognitive dysfunction *n*; %	6; 43%	25; 56.8%	0.3617	25; 80.6%	**0.0112**	28; 60.9%	0.2337	6; 40%	0.8759	84; 61.8%
Malignancy; *n*; %	2; 14%	1; 2.3%	0.1416	2; 6.5%	0.5776	2; 7%	0.2295	0; 0%	0.2241	5; 3.6%
CSF elevated cell count; *n*; %	4; 29%	8; 18.2%	0.4574	10; 32.3%	1	11; 23.9%	0.7337	4; 26.7%	1	33; 24.3%
CSF elevated protein; *n*; %	6; 43%	39; 88.6%	**0.0003**	21; 67.7%	0.1147	33; 71.7%	**0.0473**	8; 53.3%	0.5726	101; 74.3%
CSF positive OCB; *n*; %	3; 21%	6; 13.6%	0.6725	2; 6.5%	0.1656	6; 13%	0.675	1; 6.7%	0.3295	15; 11%

A comparison was made between the indicated parameters of the therapy groups and the patient group without therapy. The calculation was carried out using the Chi-Square test for patient numbers ≥ 5, or Fisher’s exact test for group sizes of ≤ 5 patients with a significance level of *p* < 0.05 (*), *p* < 0.01 (**), *p* < 0.001 (***), and *p* = 0.000 (****), p values reaching statistical significance after adjustment using the Benjamini–Hochberg procedure with a false discovery rate of 10% are indicated in bold

*CSF* Cerebrospinal fluid, *CASPR2* ontactin-Associated-Protein-like-2, *IVIG* in travenous immunoglobulins, *ivMP* intravenous methylprednisolone, *LGI1* eucine-rich-Glioma-Inactivated-1, *mRS* modified Rankin Scale, *NMDAR* N-methyl-D-aspartate receptor, *OCB* oligoclonal bands, *PE* plasma exchange

The ivMP mono group of the anti-NMDAR encephalitis group had a significantly higher incidence of movement disorder than the untreated patients. Patients in the ivMP + IVIG, ivMP + PE and triple therapy groups were found to be younger than those in the untreated group. Psychiatric symptoms, movement disorders and increased CSF cell counts were more common in the ivMP + IVIG group than in the untreated group. This was also observed in the ivMP + PE and ivMP + PE + IVIG groups, although total CSF protein was also elevated significantly more often in both groups, and epileptic seizures occurred more frequently in the ivMP + PE + IVIG group.

In the group of patients with antibodies against LGI1 or CASPR2, the ivMP mono and ivMP + PE groups had higher CSF total protein levels than the untreated group. Cognitive deficits were significantly more common in ivMP + PE than in untreated patients.

### Outcome comparisons between the therapy groups

**Fig. 1:**
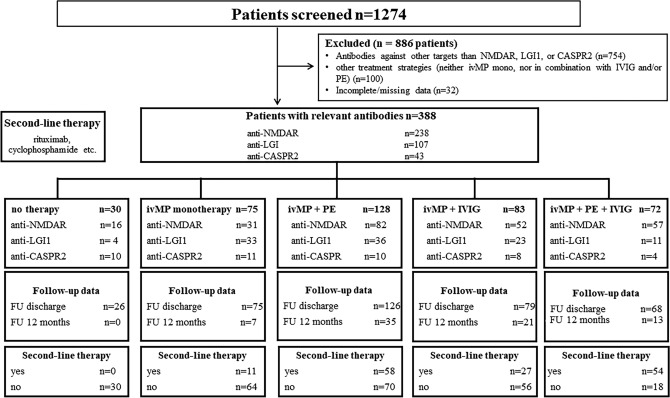
Flow chart of study design. After exclusion, 388 patients were analyzed, divided according to the respective therapy approach and the corresponding antibody. *CASPR2* contactin-associated-protein-like-2, *FU* follow-up, *IVIG* intravenous immunoglobulins, *ivMP* intravenous methylprednisolone, *LGI1* leucine-rich-glioma-inactivated-1, *mRS* modified Rankin Scale, *NMDAR* N-methyl-D-aspartate receptor, *PE* plasma exchange

Of the 238 patients diagnosed with anti-NMDAR encephalitis, 31 were treated with ivMP monotherapy, 52 with ivMP + IVIG, 82 with ivMP + PE and 57 with ivMP in combination with PE and IVIG. 16 patients did not receive immunosuppressive treatment. (Fig. 1, Table 1). The mean time between the onset of symptoms and the initiation of treatment was 15 days for all treatment groups, with no statistically significant differences observed between the groups. (Suppl. Figure 1A). In all treatment groups, high-dose corticosteroids were given for a median of five days, with no significant differences in treatment time (Suppl. Figure 2A). Both ivMP + IVIG and ivMP + PE + IVIG were treated with IVIG for a median of five days. The dosage did not differ between the two treatment groups, with a median of 30 g per day (Suppl. Figure 3A–C). Both ivMP + PE and ivMP + PE + IVIG were comparably distributed between plasmapheresis (ivMP + PE 65%, ivMP + PE + IVIG 63,5%) and immunoadsorption (ivMP + PE 35%, ivMP + PE + IVIG 36,5%) (Suppl. Figure 4A). A median of five cycles of PE was used in both treatment groups, with no significant differences observed.

The patient group with anti-LGI1/CASPR2 encephalitis included 107 patients with anti-LGI1 encephalitis and 43 patients with anti-CASPR2 encephalitis. A total of 44 patients received ivMP as monotherapy, 31 patients were treated with ivMP + IVIG, 46 with ivMP + PE, 15 patients with ivMP, PE and IVIG and 14 patients did not receive any treatment (Fig. 1, Table 1). As in anti-NMDAR encephalitis neither time till onset of treatment, duration of steroid therapy, dosage of IVIG treatment, number of PE cycles nor proportion of immunoadsorption vs. plasmapheresis differed between treatment groups (Suppl. Figures 1–4B).

**Fig. 2:**
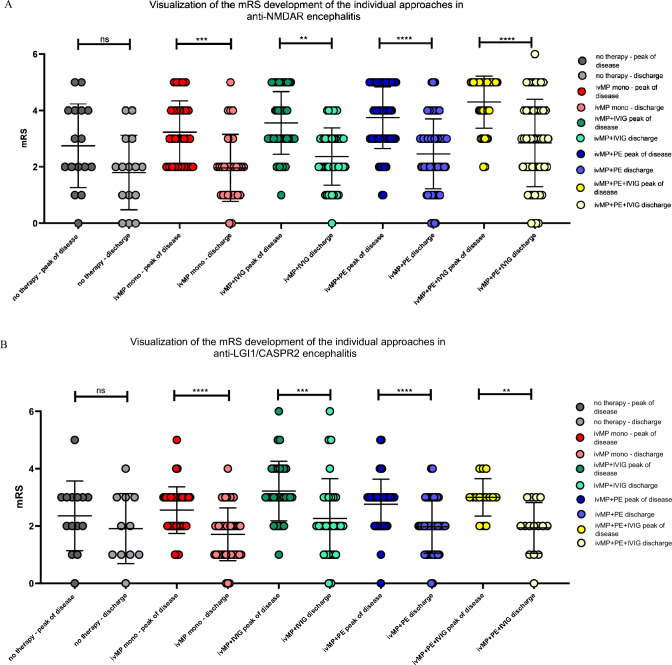
**A**, **B** Illustration of the distribution of mRS values of patients with anti-NMDAR encephalitis (**A**) and anti-LGI1/CASPR2 encephalitis (**B**) in the respective treatment groups in the course from peak of disease to time of discharge The statistical analysis was conducted using the Mann–Whitney test, with a significance level of *p* < 0.05 (*), *p* < 0.01 (**), *p* < 0.001 (***), and *p* = 0.000 (****).*CASPR2* contactin-associated-protein-like-2, *IVIG* intravenous immunoglobulins, *ivMP* intravenous methylprednisolone, *LGI1* leucine-rich-glioma-inactivated-1, *mRS* modified Rankin Scale, *NMDAR* N-methyl-D-aspartate receptor, *PE* plasma exchange

At the time of maximum disease severity, patients in the ivMP + PE + IVIG treatment group were more affected (median mRS 5; 2–5) compared to patients without therapy (median mRS 3; 0–5, *p* = 0.0026), ivMP mono (median mRS 3; 2–5, *p* = 0.0003), ivMP + IVIG (median mRS 3; 1–5, *p* = 0.0066) and compared to ivMP + PE (median mRS 4; 1–5, *p* = 0.0412). In patients diagnosed with anti-LGI1 and anti-CASPR2 encephalitis, no significant differences in mRS levels at the peak of the disease were observed between the treatment groups. (Fig. 2B) At discharge, there was a significant difference in mRS between ivMP mono (median mRS 2; 0–5) and ivMP + PE + IVIG (median mRS 3; 0–6; *p* = 0,0287) for anti-NMDAR encephalitis; for encephalitis caused by antibodies against LGI1 and CASPR2, no significant differences in mRS were seen between treatment groups. A significant mRS reduction from the peak of disease at the time of discharge was demonstrated in each treatment group (Fig. 2A, B). In untreated patients, no significant mRS reduction was observed (Fig. 2A, B).

### Disease course in the individual therapy groups

**Fig. 3:**
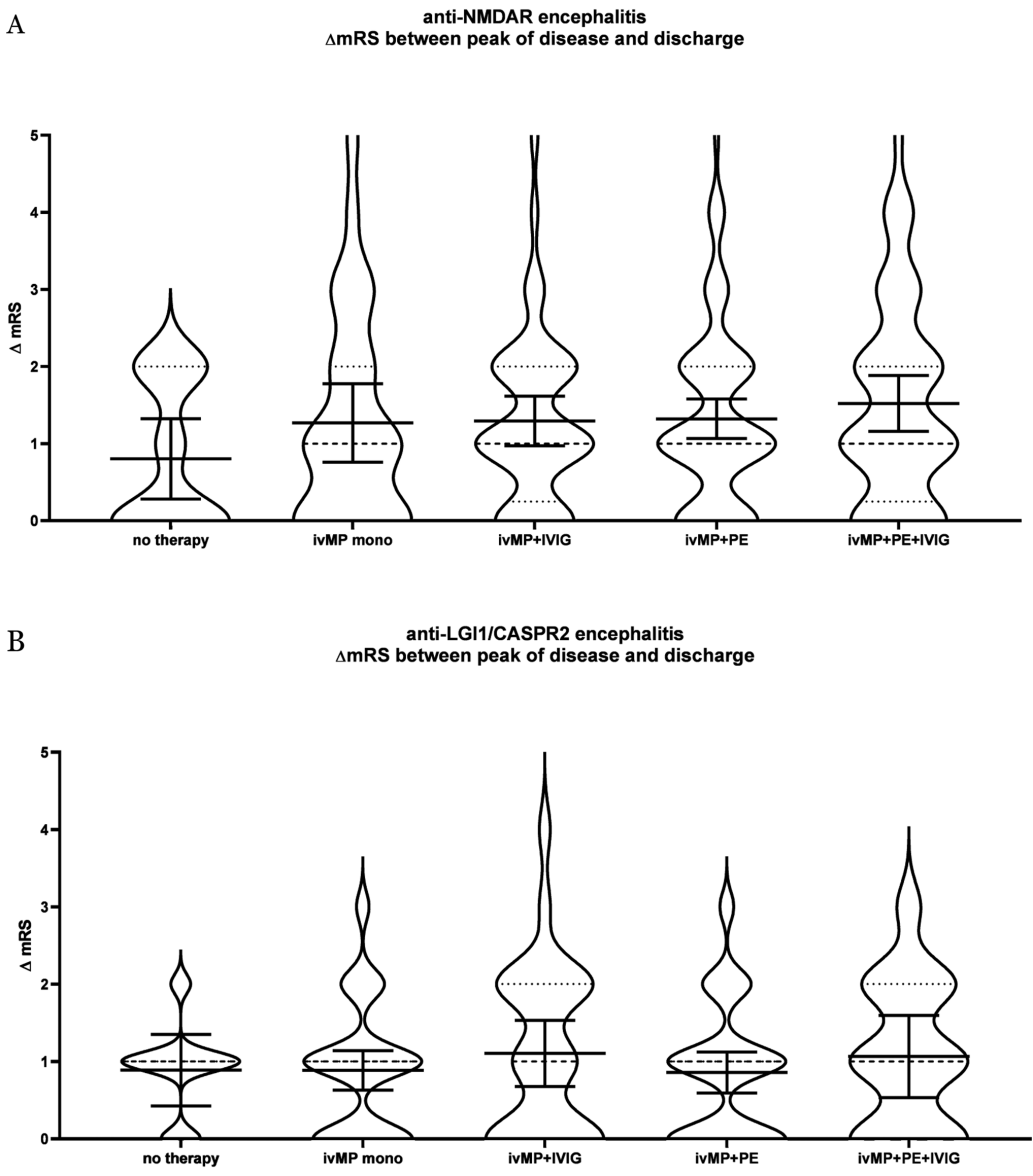
**A**, **B** Distribution of the ΔmRS between the peak of disease and time of discharge in the individual treatment approaches in the anti-NMDAR (**A**) and the anti-LGI1/CASPR2 group (**B**) including mean with 95% CI (solid line) and median (dashed line). The statistical analysis was conducted using the Kruskal–Wallis test. *CI* confidence intervall, *CASPR2* contactin-associated-protein-like-2, *FU* follow-up, *IVIG* intravenous immunoglobulins, *ivMP* intravenous methylprednisolone, *LGI1* leucine-rich-glioma-inactivated-1, *mRS* modified Rankin Scale, *NMDAR* N-methyl-D-aspartate receptor, *PE* plasma exchange

Since the ivMP + PE + IVIG in the anti-NMDAR encephalitis group had a higher mRS at the peak of the disease, the mRS reduction within the individual treatment groups was analysed. No statistically significant differences were found in ΔmRS reduction among the groups. However, for anti-NMDAR encephalitis, a trend can be observed that the mRS reduction is most pronounced for treatment with ivMP + PE + IVIG. Furthermore, the mean mRS between ivMP + IVG and ivMP + PE is almost equivalent, with a nearly identical confidence interval (95%CI) (Fig. 3A). In the anti-LGI1/CASPR2 group, there is a trend that ivMP + IVIG contributes to the strongest mRS reduction (Fig. 3B).

**Fig. 4:**
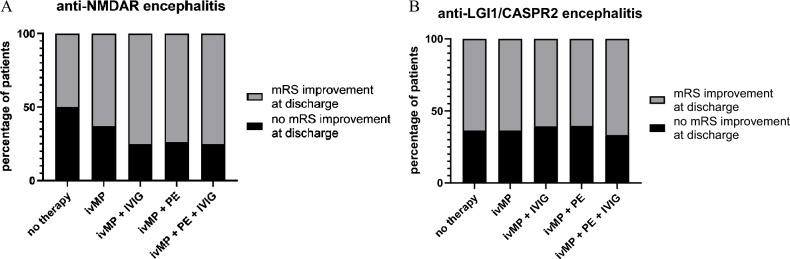
**A**, **B** Improvement in the modified Rankin Scale (mRS) score resulting from the application of individual therapeutic approaches. **A** Illustrates the ratios between non-responders and those who improved in the mRS from peak of disease to discharge in patients with anti-NMDAR encephalitis. **B** Illustrates the proportion of non-responders at discharge in patients with anti-LGI1/CASPR2 encephalitis. *CASPR2* contactin-associated-protein-like-2, *IVIG* intravenous immunoglobulins, *ivMP* intravenous methylprednisolone, *LGI1* leucine-rich-glioma-inactivated-1, *mRS* modified Rankin Scale, *NMDAR* N-methyl-D-aspartate receptor, *PE* plasma exchange

The proportion of patients suffering from anti-NMDAR encephalitis with no change in mRS was 50% in the non-treatment group, 37% in the ivMP monotherapy group, 25% for ivMP + IVIG and ivMP + PE + IVIG and 26.25% in the ivMP + PE therapy group (Fig. 4A). In the anti-LGI1/CASPR2 group, the proportions of patients without mRS improvement were more similar in the individual groups (no treatment group 36,4%, ivMP monotherapy 36.4%, ivMP + IVIG 39.3%, ivMP + PE 39.5%, ivMP + PE + IVIG 33.3%) (Fig. 4B).

A multivariate analysis was conducted to ascertain the potential influences on the clinical improvements observed in the respective therapy groups, as measured by the changes in the mRS value (ΔmRS).

In the anti-NMDAR encephalitis group, the data demonstrated that patients who received the ivMP + PE + IVIG exhibited a trend of a threefold increase in the probability of mRS improvement when compared with patients who received ivMP monotherapy. No observable influence of maintenance therapy, the presence of a teratoma or the patients’ age on the decline in mRS at discharge was identified (Table 2). In the anti-LGI1/CASPR2 group, there was a tendency for treatment with ivMP + PE + IVIG to result in a greater reduction in mRS compared to ivMP monotherapy. Furthermore, there were indications that maintenance therapy (rituximab, cyclophosphamide) and treatment of a proven neoplasm contributed to an improvement in mRS (Table 3).

**Table 2:** Multivariate analysis of the outcome for the anti-NMDAR encephalitis group

Parameter	*p*	DF	OR	95% CL
**Therapy**				
ivMP + IVIG vs. ivMP	0.067	1	2.34	−0.59–1.762
ivMP + PE vs. ivMP	0.302	1	1.64	−0.443–1.431
ivMP + PE + IVIG vs. ivMP	0.012	1	3.35	0.266–2.150
**Time to therapy initiiation (*d*)**	0.067	1		
≤ 28 vs. ≥ 28			1.68	−0.037–1.079
**Maintenance therapy**	0.219	1		
Yes vs. no			0.72	−0.844–0.193
**Removed teratoma**	0.401	1		
Yes vs. no			1.26	−0.307–0.769
**Age**	0.478	1	1.01	−0.25–0.012

Illustration of the potential influences on the ΔmRS (mRS difference between the peak of disease and discharge) and their corresponding odds ratios in patients with anti-NMDAR encephalitis. The calculation was performed using ordinal logistic regression

Unadjusted *p* values are indicated. *p* values reaching statistical significance after adjustment using the Benjamini–Hochberg procedure with a false discovery rate of 10% are indicated in bold

*DF* Degrees of Freedom, *IVIG* Intravenous immunoglobulins, *ivMP* intravenous methylprednisolone, *mRS* Modified Rankin Scale, *NMDAR* N-methyl-D-aspartate receptor, *OR* odds ratio, *PE* plasma exchange

**Table 3:** Multivariate analysis of the outcome for the anti-LGI1/-CASPR2 encephalitis group

Parameter	*p*	DF	OR	95% CL
**Therapy**				
ivMP + IVIG vs. ivMP	0.593	1	1.42	−0.926–1.622
ivMP + PE vs. ivMP	0.551	1	1.44	−0.836–1.568
ivMP + PE + IVIG vs. ivMP	0.021	1	8.86	0.326–4.035
**Time to therapy initiiation (*d*)**	0.09	1	1.02	−0.035
**Maintenance therapy**	0.004	1		
Yes vs. no			5.233	0.516–2.793
**Treated malignancy**	0.04	1		
Yes vs. no			13.86	0.119–5.14
**Age**	0.654	1	1.01	−0.044–0.027

Illustration of the potential influences on the ΔmRS (mRS difference between the peak of disease and discharge) and their corresponding odds ratios in patients with anti-LGI1/CASPR2 encephalitis. The calculation was performed using ordinal logistic regression

Unadjusted *p* values are indicated. *p* values reaching statistical significance after adjustment using the Benjamini–Hochberg procedure with a false discovery rate of 10% are indicated in bold

*CASPR2* contactin-associated-protein-like-2, *DF* degrees of freedom, *IVIG* intravenous immunoglobulins, *ivMP* intravenous methylprednisolone, *LGI1* leucine-rich-glioma-inactivated-1, *mRS* modified Rankin Scale, *OR* odds ratio, *PE* plasma exchange

**Fig. 5:**
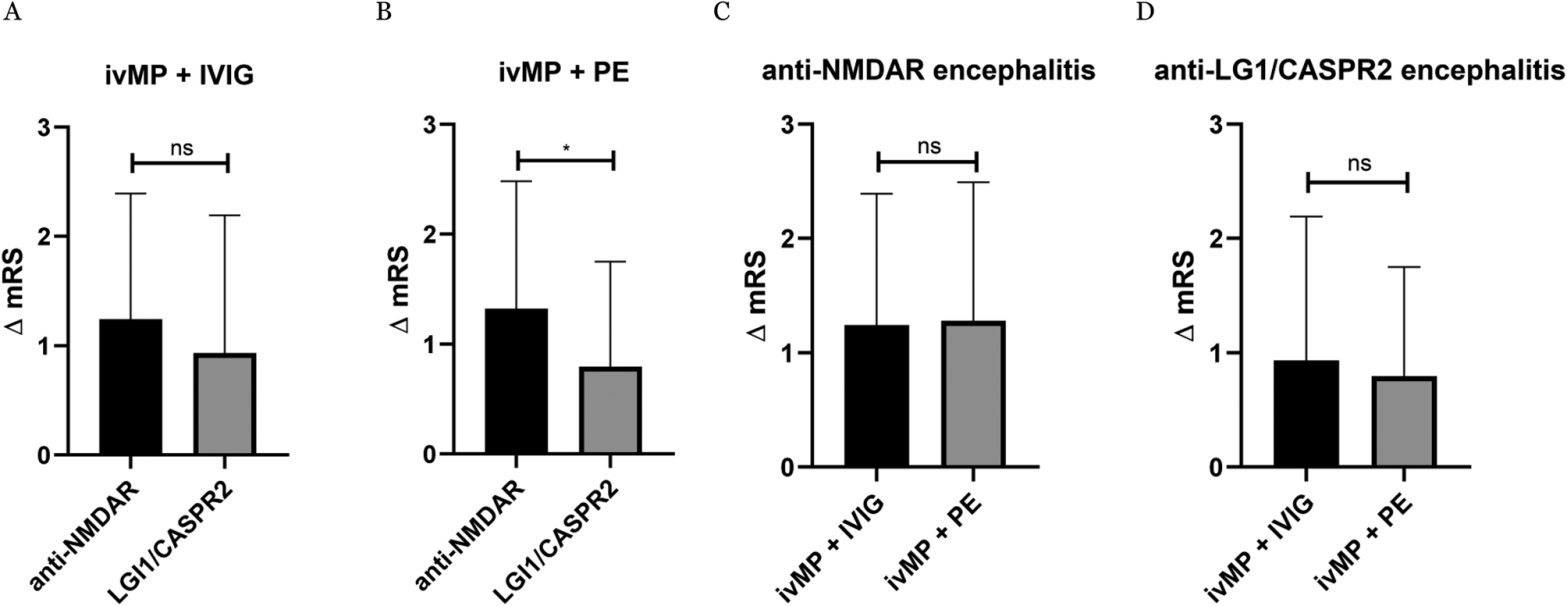
Comparisons of mRS differences within the respective therapy groups ivMP + IVIG and ivMP + PE between the detected antibodies against NMDAR vs. LGI1/CASPR2. The mRS difference is significantly higher in the ivMP + PE group compared to patients with antibodies against LGI1/CASPR2 (*p* = 0.032) (**B**). This difference cannot be detected in the ivMP + IVIG group (**A**). In addition, the subdivision was made according to antibody detection (anti-NMDAR vs. anti-LGI1/CASPR2). The subgroups were compared according to therapy (ivMP + IVIG vs ivMP + PE). There were no differences by therapy neither for anti-NMDAR nor anti-LGI1/CASPR2 (C, D). The Mann–Whitney test was used for the analysis. (*p* < 0.05 *, *p* < 0.01 **, *p* < 0.001 ***, *p* = 0.000 ****). *CASPR2* contactin-associated-protein-like-2, *IVIG* intravenous immunoglobulins, *ivMP* intravenous methylprednisolone, *LGI1* leucine-rich-glioma-inactivated-1, *mRS* modified Rankin Scale, *NMDAR* N-methyl-D-aspartate receptor, *PE* plasma exchange

A direct comparison of the treatment options ivMP + IVIG and ivMP + PE demonstrated that patients with anti-NMDAR antibodies who were treated with ivMP + PE exhibited a significantly greater reduction in the mRS than anti-LGI1/CASPR2 patients treated with ivMP + PE compared to patients with anti-LGI1 or anti-CASPR2 antibodies (*p* = 0.032), (Fig. 5B). We did not observe this difference between the groups when patients were treated with ivMP + IVIG (*p* = 0.68), (Fig. 5A).

No difference in treatment response between ivMP + IVIG and ivMP + PE was seen among patients with anti-NMDAR antibodies. Also, in patients with anti-LGI1/CASPR2 antibody-mediated encephalitis, ivMP + IVIG or ivMP + PE treatment did not result in a significantly different mRS outcome (anti-NMDAR *p* = 0.80; Fig. 5C; anti-LGI1/CASPR2 *p* = 0.38; Fig. 5D).

**Fig. 6:**
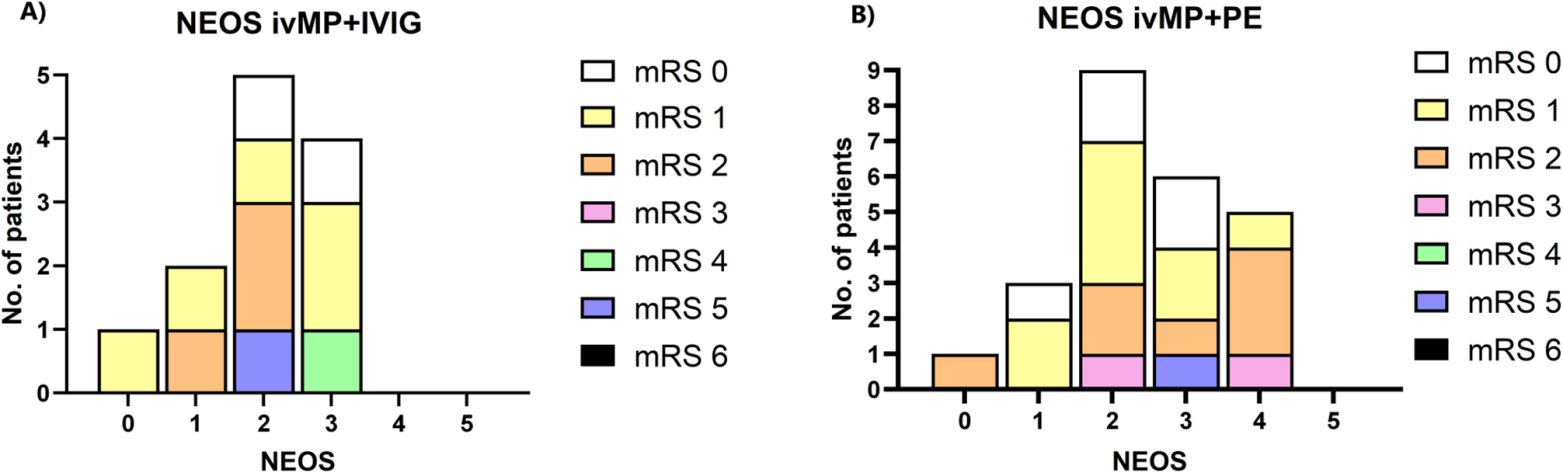
**A**, **B** The following illustrations depict mRS values at 12 months post-disease onset, according to the NEOS score, for patients with anti-NMDAR encephalitis who received ivMP + IVIG (6A) and those who received ivMP + PE (6B). In both treatment groups, the outcomes were exclusively favourable, with mRS of 0–2 for NEOS values of 0–1. Even with higher NEOS values of 2–4, the outcome was predominantly favourable, with mRS 0–2. *IVIG* Intravenous immunoglobulins, *ivMP* intravenous methylprednisolone, *mRS* modified Rankin Scale, *NEOS* anti-NMDAR encephalitis One-Year Functional Status, *NMDAR* N-methyl-D-aspartate receptor, *PE* plasma exchange

Our findings demonstrated that both patient groups treated with ivMP + IVIG and ivMP + PE with an N-methyl-D-aspartate Encephalitis One-Year Functional Status Score (NEOS score) of 0–1 exhibited exclusively favourable outcomes, with an mRS of 0–2. However, patients with NEOS 2–4 also demonstrated predominantly favourable outcomes, with an mRS of 0–2 (Fig. 6A, B).

## Methods

### Study population

All patient data were retrieved from the GENERATE registry, in which patients were included after written informed consent by themselves or their legal representatives. The network consists of 112 centers from Germany, Austria and Switzerland and acts as a pro- and retrospective register for data on the clinical course of patients with AE. Patients enrolled between 2014 and June 2020 were included.

The analysis was conducted as a multicenter study with data from a total of 45 participating centers. The individual centers contributed data from one to 62 patients. The data was entered into the register by experienced clinicians and scientists, who did so in accordance with the clinical documentation of the individual centres. An additional quality assurance measure was implemented in the form of a plausibility check of the entered data by medical documentarists.

The present study included only patients who, based on clinical, laboratory, electrophysiological and radiological findings, fulfilled the criteria for a definite autoimmune encephalitis according to Graus criteria [12]. Furthermore, patients were required to have received treatment with high-dose corticosteroids (0.5–1 g per single dose; treatment duration of three to five days) during the acute phase of the disease, as monotherapy or in combination with IVIG, PE or both. In addition, the analysis was expanded to include a group of patients who did not receive any therapy. Following the screening of 1274 patients, 520 patients were found to have antibodies only to either the NMDA receptor, LGI1 or CASPR2, while 754 patients had other auto-antibodies. Patients with more than one antibody (e.g. –anti-NMDAR and anti-IgLON5) were excluded from the study. Furthermore, 100 patients who received other treatment strategies, not including ivMP monotherapy or combinations of ivMP + IVIG, ivMP + PE, or all three treatments, had to be excluded from the study, although we included patients who remained completely untreated to make the best possible comparisons. 32 patients with incomplete documentation of primary outcome data were also excluded.

The final number of patients included was *n* = 388 (Fig. 1).

Only adult patients who had reached the age of 18 were included. For the purposes of the calculations, patients with anti-NMDAR encephalitis were considered separately, with those exhibiting IgG1-mediated disease and those exhibiting anti-LGI1/CASPR2 encephalitis pooled according to their IgG4 pathology. A comparison was made of the clinical conditions (mRS) between the treatment groups both at the peak of disease and at discharge, as well as the outcome of treated patients after 12 months. Potential influences on the mRS reduction at discharge were analyzed, and the individual antibody groups and their mRS reduction were also compared depending on the therapy carried out (ivMP + IVIG vs. ivMP + PE).

All aspects of the study have been conducted in accordance with the declaration of Helsinki.

Ethical review and approval were not required for the study on human participants according to local legislation and institutional requirements. The patient himself or a legal guardian of the patient gave written informed consent to participate in this study.

### Clinical parameter

Blood and CSF were examined at local accredited laboratories, using a cell-based assay for the detection of antibodies against NMDAR, LGI1 and CASPR2 (Euroimmun, Lübeck). CSF white cell count, protein level and oligoclonal bands were analyzed. White blood cell count ≥ 5/µl was defined as CSF pleocytosis and CSF protein levels > 0.5 g/l were defined as elevated. Clinical outcome and disease course were analyzed by comparing the modified Rankin Scale (mRS) between the peak of disease and the time of discharge. “Peak of disease” refers to the most severe symptoms of encephalitis at the time of disease manifestation. The mRS at the time of discharge was defined as the primary outcome parameter. In addition, the ‘anti-NMDAR Encephalitis One-Year Functional Status score’ (NEOS score) for each patient with antibodies against NMDAR was assessed. NEOS score is defined by the presence of an increased cell count in CSF (> 20/µl), abnormalities in cranial MRI, initiation of therapy after more than 4 weeks after symptom onset, lack of clinical improvement after 4 weeks, and the need for intensive care. A low score is associated with a low mRS, which is indicative of a favorable outcome [17]. As the NEOS score was not explicitly requested in the data query in the GENERATE register, it was calculated from the available information as part of the data analysis.

### Statistical analysis

We used SPSS (IBM® SPSS® Statistics 27, Armonk, New York, USA) for statistical tests and calculations. Graphics were created with Prism Software (GraphPad Prism 8, California, USA).

Two-tailed *p*-values of *p < 0.05, **p < 0.01, ***p < 0.001 and ****p < 0.0001 were considered significant and designated accordingly. Kruskal–Wallis test followed by Dunn’s multiple comparisons test was used to compare ordinal or continuous variables with > two subgroups, for two subgroups the Mann–Whitney test was used. Chi-Square test was used to compare categorical variables, and Fisher’s exact test was applied when expected frequencies were below 5. Multivariate analysis was performed by ordinal logistic regression.

## Discussion

This study investigated the outcomes of patients with AE caused by the three most antibodies (anti-NMDAR, anti-LGI1 and anti-CASPR2) with respect to IVIG treatment in a large cohort. In all four treatment groups, we observed a significant reduction in disease severity during follow-up. Untreated patients in the anti-NMDAR encephalitis group as well as in the anti-LGI1/CASPR2 group did not show a significant improvement in mRS at the time of discharge.

The analysis revealed no significant differences in mRS improvement between patients treated with ivMP + IVG and those treated with ivMP + PE at any time point, irrespective of the antibody present. The results of our study indicate that there is no evidence to suggest that the treatment of autoimmune encephalitis with high-dose corticosteroids and PE is either more or less effective than treatment with high-dose corticosteroids and IVIG. This is in line with most other observations comparing IVIG and PE in patients with different neuroimmunological diseases. In a randomized controlled trial involving 84 patients with moderate to severe forms of myasthenia gravis, the equivalence of IVIG and PE was shown [18]. In a meta-analysis of 23 reports on outcomes of patients with different neuroimmunological diseases, the effects of IVIG treatment are considered equal to those of PE [19]. A review of 44 studies on different autoimmune diseases in neurology could not establish the superiority of one procedure over the other when comparing the use of IVIG against PE [20].

Nevertheless, in myasthenia gravis it was demonstrated that the duration of treatment in the intensive care unit under PE was shorter and that the patients achieved significantly fewer points in the scores (QMG, MMT, ADL) after the end of ventilation than under IVIG therapy [21, 22]. Patients with antibodies against the NMDAR showed a significantly higher decrease in mRS difference compared to patients with anti-LGI1/CASPR2 antibodies when treated with ivMP + PE. The data suggest that patients with anti-NMDAR antibodies might benefit from plasma exchange compared to anti-LGI1- and anti-CASPR2 encephalitis.

This could be related to the different immunoglobulin subclasses. A poorer response of IgG4-mediated immunopathies to PE has already been described [23].

A small group of patients could be identified within the dataset for both antibody groups who did not receive any immunotherapy, neither primary nor secondary therapy. Unfortunately, the documentation is insufficient for a statement on the clinical status after 12 months, possibly because patients who did not receive any therapy for whatever reason, e.g., because they refused it, also did not attend any follow-up appointments. Despite the absence of substantial mRS improvements in patients who did not receive therapy, a modest downward trend in mRS scores at the time of discharge was observed. This can be attributed to the natural progression of the disease, as well as the potential benefits of supportive interventions, such as anti-seizure medication or antipsychotics. The limited sample size and the high variability of the results prevent the drawing of reliable conclusions regarding the systematic nature of the observed differences between the untreated subgroups. The conduction of further studies with larger samples is necessary to reliably assess possible effects.

In patients diagnosed with anti-NMDAR encephalitis, a notable discrepancy in disease severity at its peak was observed across the treatment groups. The hypothesis that patients presenting with more severe symptoms were more likely to receive an intensive combination of treatments, while patients exhibiting milder symptoms may be treated with steroids alone is therefore supported by these findings. This therapeutic approach likely reflects clinical judgment based on symptom severity rather than a strictly standardized protocol. However, this approach introduces a selection bias, as the differential treatment allocation may confound the interpretation of treatment outcomes. Within the treatment groups, the ivMP monotherapy group exhibited the highest proportion of non-responders; however, an even higher proportion of patients demonstrated a lack of improvement in mRS in the untreated patient group. While the differences observed were not statistically significant, it is crucial to acknowledge the impact of the varying group sizes on the calculations’ results. It is notable that, initially, the mRS values differed between the groups, but equalized over a year.

It should be noted that the number of patients with complete documentation continues to decrease over time, which may potentially diminish the significance of the data.

In the anti-NMDAR encephalitis group, a trend for a clearer mRS reduction was observed with ivMP + PE + IVIG compared to ivMP monotherapy. In patients with encephalitis caused by antibodies against LGI1/CASPR2, in addition to the trend for mRS improvement with ivMP + PE + IVIG compared to ivMP monotherapy, there was also a hint of mRS improvement with the application of maintenance therapy involving rituximab or cyclophosphamide and the treatment of an existing neoplasm. However, the 95% confidence interval indicates a considerable degree of uncertainty with regard to the effect size. This is assumed to be partly caused by an inhomogeneity of the sizes of the treatment groups. The untreated patients were excluded from the multivariate analysis in both the anti-NMDAR encephalitis and the anti-LGI1/CASPR2 encephalitis groups due to their even smaller and therefore even more inhomogeneous size. Otherwise, a further destabilization of the model would have had to be feared, which would have further reduced the robustness of the findings. In our work, we compared the predicted outcomes using the NEOS score with the actual mRS of the patients after 12 months. In both treatment groups, ivMP + IVIG and ivMP + PE, NEOS 0–1 demonstrated exclusively favorable outcomes at the 12-month follow-up. Even if NEOS values were higher, positive outcomes were largely achieved in both treatment groups. The predicted results and the actual mRS after 12 months also support the assumption that none of the treatment methods can be regarded as superior or inferior concerning the outcome based on the NEOS score. The discrepancy between the expected and the actual measured mRS value after 12 months of FU, based on the calculated NEOS score, could be attributed to the increasing expertise of the participating treatment centres. A limitation of our study is that follow-up data were only available from a subgroup of patients and the number of cases became even smaller with further splitting of the groups, which limits the conclusions at 12-month follow-up. Despite the fact that components of the data collection were executed prospectively, the present study is predominantly a retrospective analysis of outcomes based on the treatments administered. Regrettably, a sufficiently large control group of untreated patients is not available, which limits the possibility of a comprehensive assessment of the natural course of the disease or a direct comparison of the efficacy of different therapeutic approaches. This absence of a robust control cohort represents a significant limitation, which may have a potential impact on the overall generalizability and interpretability of the findings. Despite the lack of randomization, no significant differences were observed between the individual treatment groups within the antibody-specific cohorts with regard to steroid doses, IVIG doses and duration of application or the type and duration of treatment with PE.

However, there was no standardized treatment protocol, which represents a limitation when considering the impact of the comparison between IVIG and PE treatment on the outcome, given that the dose and duration were not standardized. A prospective, randomized. Multicenter observational study should be conducted to further evaluate the efficacy of IVIG patients with autoimmune encephalitis. Study participants will receive a standardized IVIG therapy at a dosage of 2 g/kg body weight over five days, with the possibility of repeat administration based on clinical assessment. The primary endpoint of the study is clinical improvement, measured using the Clinical Assessment Scale for Autoimmune Encephalitis score (CASE score) at discharge as well as at three and six months. Secondary endpoints may also include changes in neurocognitive parameters and relapse frequency. In principle, all known autoantibodies associated with AE could be considered, with the analysis stratified by antibody subtype to enhance the interpretability of results for individual AE subtypes.

A notable limitation pertains to the clinical status of patients in this study, which was represented semi-quantitatively using the modified Rankin Scale. It should be noted that this score was originally developed for stroke patients and is not specifically tailored for describing the condition of patients with autoimmune encephalitis. The more precise and disease-specific CASE score was gradually integrated into the GENERATE database but unfortunately was not available for patients assessed by us in 2020 [24]. Due to the complex nature of the CASE score and its numerous components, retrospective calculation was not feasible. It is acknowledged that the implementation of the CASE score would facilitate a more precise and meaningful evaluation; however, future studies investigating the course of autoimmune encephalitis should prioritize its integration. Notwithstanding, it is noteworthy that several significant studies on autoimmune encephalitis have been conducted using mRS values, thereby demonstrating its applicability despite its inherent limitations [25–27].

Notably, it is crucial to underscore that the conclusions derived from this study exclusively pertain to patients with encephalitis caused by antibodies against NMDAR, LGI1, or CASPR2.

## Conclusion

We draw the following conclusions from our work: The efficacy of IVIG and PE additional to ivMP in patients with anti-NMDAR, -LGI1 and -CASPR2 encephalitis appears to be comparable.

Patients with anti-NMDAR antibodies have a significantly better mRS improvement with PE than patients with antibodies against LGI1 or CASPR2. Furthermore, a combination treatment with ivMP + PE + IVIG, as well as maintenance therapy with agents such as rituximab or cyclophosphamide, has a positive impact on mRS decrease at the time of discharge.

## Supplementary Information

Below is the link to the electronic supplementary material.

Supplementary file1 (TIF 132 KB)

Supplementary file2 (TIF 131 KB)

Supplementary file3 (TIF 114 KB)

Supplementary file4 (TIF 113 KB)

Supplementary file5 (TIF 99 KB)

Supplementary file6 (TIF 101 KB)

Supplementary file7 (TIF 95 KB)

Supplementary file8 (TIF 97 KB)

Supplementary file9 (TIF 101 KB)

Supplementary file10 (TIF 94 KB)

Supplementary file11 (TIF 110 KB)

Supplementary file12 (TIF 111 KB)

## Data Availability

All data generated or analysed in this study are included in this published article and its supplemental information files.

## Abbreviations

ADL: Activities of daily living
AE: Autoimmune encephalitis
CASE score: Clinical Assessment Scale for Autoimmune Encephalitis score
CASPR2: Contactin-associated-protein-like-2
CI: Confidence interval
CIDP: Chronic inflammatory demyelinating polyneuropathy
CNS: Central nervous system
CSF: Cerebrospinal fluid
DF: Degree of freedom
FU: Follow up
GENERATE: German Network for Research on AuToimmune Encephalitis
Ig: Immunoglobulin
IVIG: Intravenous immunoglobulins
ivMP: Intravenous methylprednisolone
LGI1: Leucine-rich-glioma-inactivated-1
MMT: Manual muscle test
mRS: Modified Rankin Scale
NMDAR: N-methyl-D-aspartate receptor
NEOS: Anti-NMDAR encephalitis one-year functional status
NHS: National health service
OCB: Oligoclonal bands
PE: Plasma exchange
QMG: Quantitative Myasthenia Gravis Score
Suppl.: Supplementary

## References

[1] Dalmau J (2016) NMDA receptor encephalitis and other antibody-mediated disorders of the synapse: the 2016 cotzias lecture. Neurology 87(23):2471–2482 27920282 10.1212/WNL.0000000000003414 PMC5177671

[2] Leypoldt F, Wandinger KP, Bien CG, Dalmau J (2013) Autoimmune encephalitis. Eur Neurol Rev 8(1):31–37 27330568 10.17925/ENR.2013.08.01.31 PMC4910513

[3] van Sonderen A, Arino H, Petit-Pedrol M, Leypoldt F, Kortvelyessy P, Wandinger KP et al (2016) The clinical spectrum of Caspr2 antibody-associated disease. Neurology 87(5):521–528 27371488 10.1212/WNL.0000000000002917 PMC4970662

[4] Graus F, Titulaer MJ, Balu R, Benseler S, Bien CG, Cellucci T et al (2016) A clinical approach to diagnosis of autoimmune encephalitis. Lancet Neurol 15(4):391–404 26906964 10.1016/S1474-4422(15)00401-9 PMC5066574

[5] Abboud H, Probasco JC, Irani S, Ances B, Benavides DR, Bradshaw M et al (2021) Autoimmune encephalitis: proposed best practice recommendations for diagnosis and acute management. J Neurol Neurosurg Psychiatry 92(7):757–768 33649022 10.1136/jnnp-2020-325300 PMC8223680

[6] Rodriguez A, Klein CJ, Sechi E, Alden E, Basso MR, Pudumjee S et al (2022) LGI1 antibody encephalitis: acute treatment comparisons and outcome. J Neurol Neurosurg Psychiatry 93(3):309–315 34824144 10.1136/jnnp-2021-327302 PMC8862031

[7] Guo Y, Tian X, Wang X, Xiao Z (2018) Adverse effects of immunoglobulin therapy. Front Immunol 9:1299 29951056 10.3389/fimmu.2018.01299 PMC6008653

[8] Hamrock DJ (2006) Adverse events associated with intravenous immunoglobulin therapy. Int Immunopharmacol 6(4):535–542 16504916 10.1016/j.intimp.2005.11.015

[9] Imbach P, Barandun S, d’Apuzzo V, Baumgartner C, Hirt A, Morell A et al (1981) High-dose intravenous gammaglobulin for idiopathic thrombocytopenic purpura in childhood. Lancet 1(8232):1228–1231 6112565 10.1016/s0140-6736(81)92400-4

[10] Stohl W, Elliot JE (1996) In vitro inhibition by intravenous immunoglobulin of human T cell-dependent B cell differentiation induced by staphylococcal superantigens. Clin Immunol Immunopathol 79(2):122–133 8620618 10.1006/clin.1996.0059

[11] Saoudi A, Hurez V, de Kozak Y, Kuhn J, Kaveri SV, Kazatchkine MD et al (1993) Human immunoglobulin preparations for intravenous use prevent experimental autoimmune uveoretinitis. Int Immunol 5(12):1559–1567 8312226 10.1093/intimm/5.12.1559

[12] Ballow M (2014) Mechanisms of immune regulation by IVIG. Curr Opin Allergy Clin Immunol 14(6):509–515 25337683 10.1097/ACI.0000000000000116

[13] Lunemann JD, Quast I, Dalakas MC (2016) Efficacy of intravenous immunoglobulin in neurological diseases. Neurotherapeutics 13(1):34–46 26400261 10.1007/s13311-015-0391-5 PMC4720677

[14] Gelfand EW (2013) Intravenous immune globulin in autoimmune and inflammatory diseases. N Engl J Med 368(8):777 23425181 10.1056/NEJMc1215489

[15] Lunemann JD, Nimmerjahn F, Dalakas MC (2015) Intravenous immunoglobulin in neurology–mode of action and clinical efficacy. Nat Rev Neurol 11(2):80–89 25561275 10.1038/nrneurol.2014.253

[16] Kinsella JA, Irani SR, Hollingsworth R, O’Shaughnessy D, Kane P, Foster M et al (2018) Use of intravenous immunoglobulin for the treatment of autoimmune encephalitis: audit of the NHS experience. JRSM Open 9(9):2054270418793021 30202534 10.1177/2054270418793021 PMC6122256

[17] Balu R, McCracken L, Lancaster E, Graus F, Dalmau J, Titulaer MJ (2019) A score that predicts 1-year functional status in patients with anti-NMDA receptor encephalitis. Neurology 92(3):e244–e252 30578370 10.1212/WNL.0000000000006783 PMC6340387

[18] Barth D, Nabavi Nouri M, Ng E, Nwe P, Bril V (2011) Comparison of IVIg and PLEX in patients with myasthenia gravis. Neurology 76(23):2017–2023 21562253 10.1212/WNL.0b013e31821e5505 PMC3109880

[19] Morales-Ruiz V, Juarez-Vaquera VH, Rosetti-Sciutto M, Sanchez-Munoz F, Adalid-Peralta L (2022) Efficacy of intravenous immunoglobulin in autoimmune neurological diseases. Literature systematic review and meta-analysis. Autoimmun Rev 21(3):103019 34920107 10.1016/j.autrev.2021.103019

[20] Pinto AA, De Seze J, Jacob A, Reddel S, Yudina A, Tan K (2023) Comparison of IVIg and TPE efficacy in the treatment of neurological disorders: a systematic literature review. Ther Adv Neurol Disord 16:17562864231154306 37006460 10.1177/17562864231154306 PMC10064470

[21] Wang Y, Huan X, Jiao K, Jiang Q, Goh LY, Shi J et al (2022) Plasma exchange versus intravenous immunoglobulin in AChR subtype myasthenic crisis: a prospective cohort study. Clin Immunol 241:109058 35690385 10.1016/j.clim.2022.109058

[22] Liu JF, Wang WX, Xue J, Zhao CB, You HZ, Lu JH et al (2010) Comparing the autoantibody levels and clinical efficacy of double filtration plasmapheresis, immunoadsorption, and intravenous immunoglobulin for the treatment of late-onset myasthenia gravis. Ther Apher Dial 14(2):153–160 20438536 10.1111/j.1744-9987.2009.00751.x

[23] Dalakas MC (2022) IgG4-mediated neurologic autoimmunities: understanding the pathogenicity of IgG4, ineffectiveness of IVIg, and long-lasting benefits of anti-B cell therapies. Neurol Neuroimmunol Neuroinflamm. 10.1212/NXI.0000000000001116 34845096 10.1212/NXI.0000000000001116 PMC8630661

[24] Lim JA, Lee ST, Moon J, Jun JS, Kim TJ, Shin YW et al (2019) Development of the clinical assessment scale in autoimmune encephalitis. Ann Neurol 85(3):352–358 30675918 10.1002/ana.25421

[25] Zhang Y, Tu E, Yao C, Liu J, Lei Q, Lu W (2021) Validation of the clinical assessment scale in autoimmune encephalitis in chinese patients. Front Immunol 12:796965 34975905 10.3389/fimmu.2021.796965 PMC8718556

[26] Thaler FS, Zimmermann L, Kammermeier S, Strippel C, Ringelstein M, Kraft A et al (2021) Rituximab treatment and long-term outcome of patients with autoimmune encephalitis: real-world evidence from the GENERATE registry. Neurol Neuroimmunol Neuroinflamm 8(6):e1088 34599001 10.1212/NXI.0000000000001088 PMC8488759

[27] Halliday A, Duncan A, Cheung M, Boston RC, Apiwattanakul M, Camacho X et al (2022) Second-line immunotherapy and functional outcomes in autoimmune encephalitis: a systematic review and individual patient data meta-analysis. Epilepsia 63(9):2214–2224 35700069 10.1111/epi.17327 PMC9796249

