# Supplementary figures and images for: Intravenous immunoglobulin as first-line acute treatment in adults with autoimmune encephalitis caused by antibodies to NMDAR, LGI1 and CASPR2
Source: J Neurol. 2025 Mar 25;272(4):287. doi: 10.1007/s00415-025-13032-0 (PMC11937155; doi:10.1007/s00415-025-13032-0)

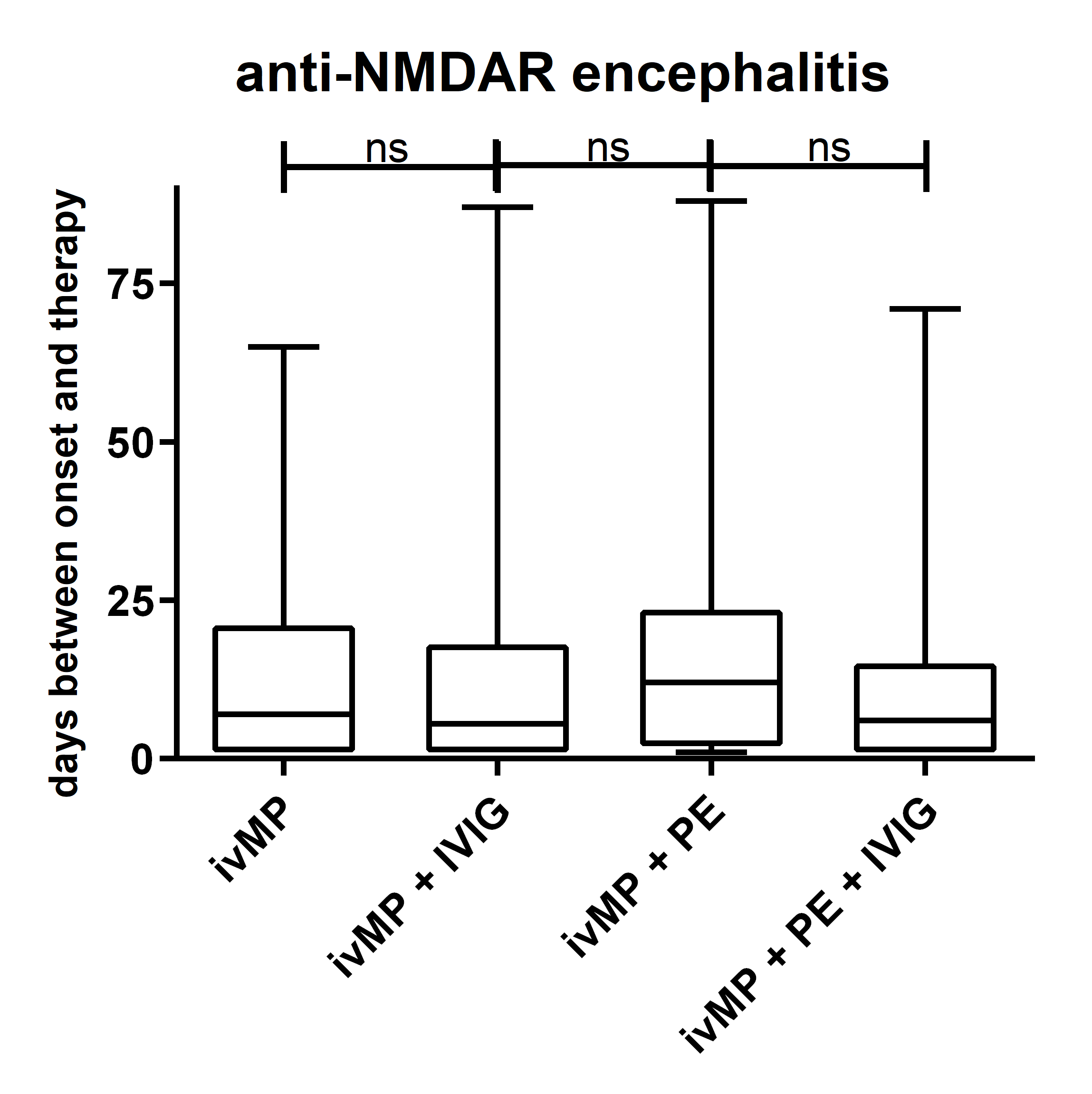

Supplement: Supplementary file 1 — Supplementary file1 (TIF 132 KB) [file 415_2025_13032_MOESM1_ESM.tif]

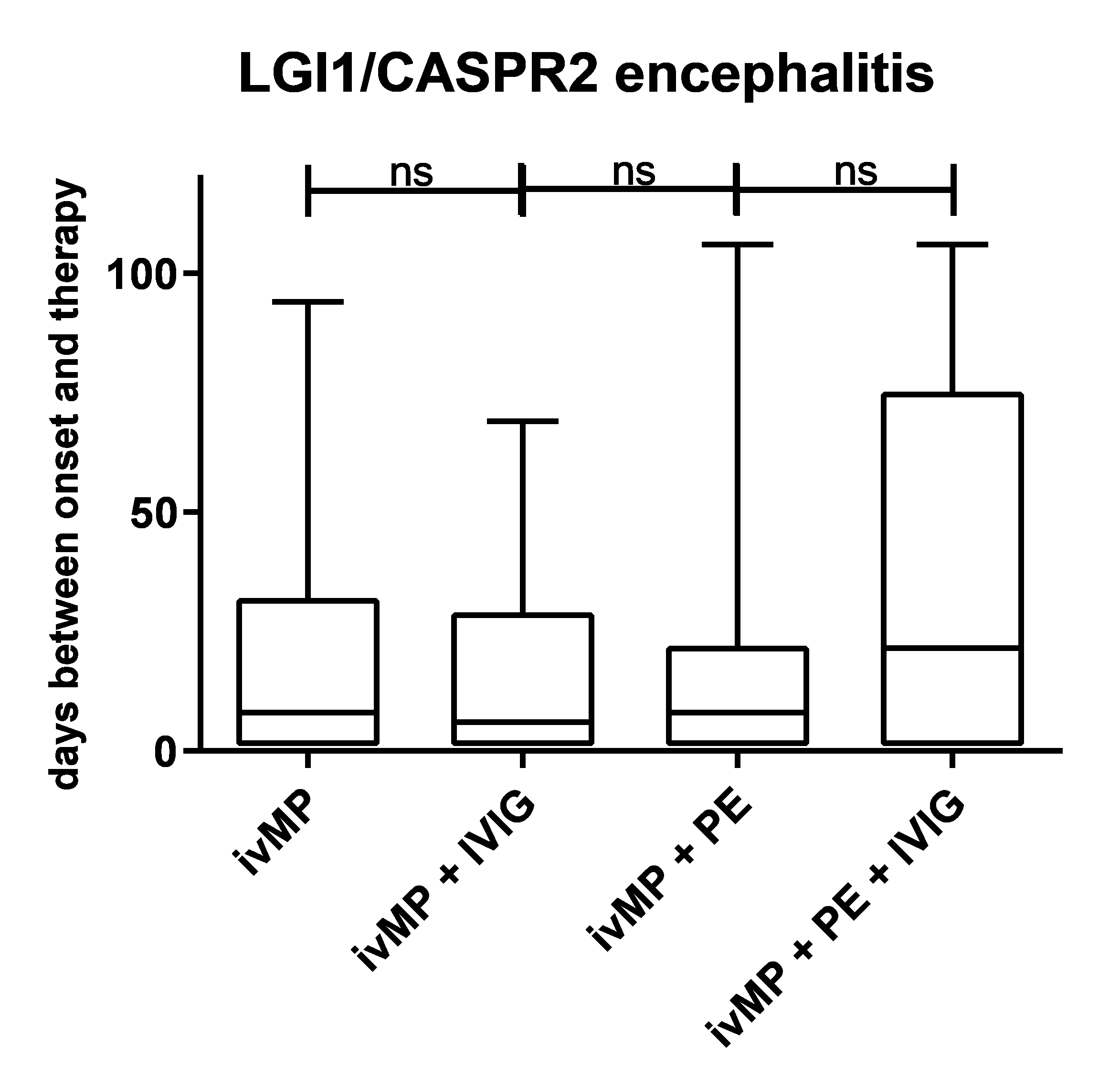

Supplement: Supplementary file 2 — Supplementary file2 (TIF 131 KB) [file 415_2025_13032_MOESM2_ESM.tif]

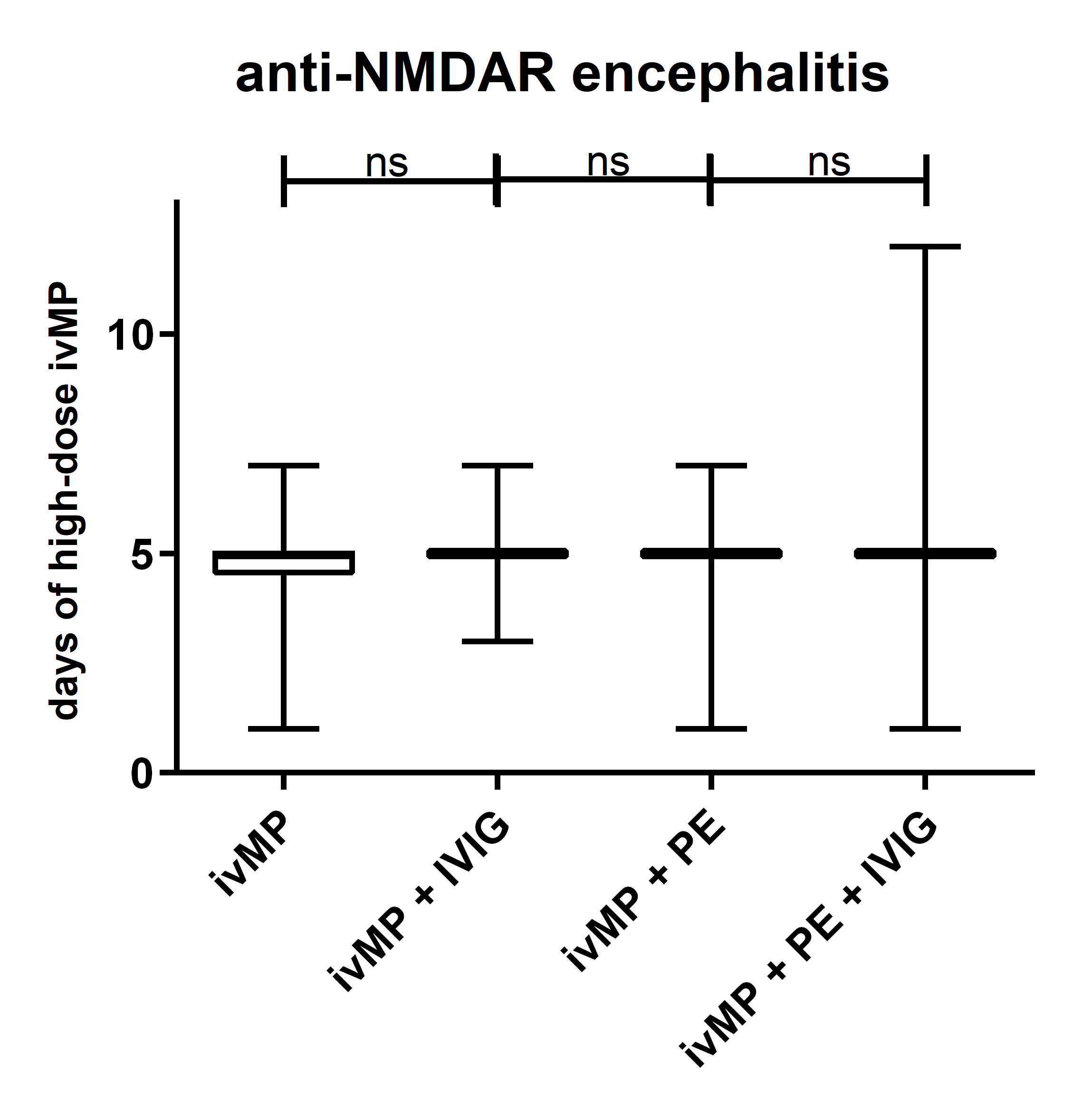

Supplement: Supplementary file 3 — Supplementary file3 (TIF 114 KB) [file 415_2025_13032_MOESM3_ESM.tif]

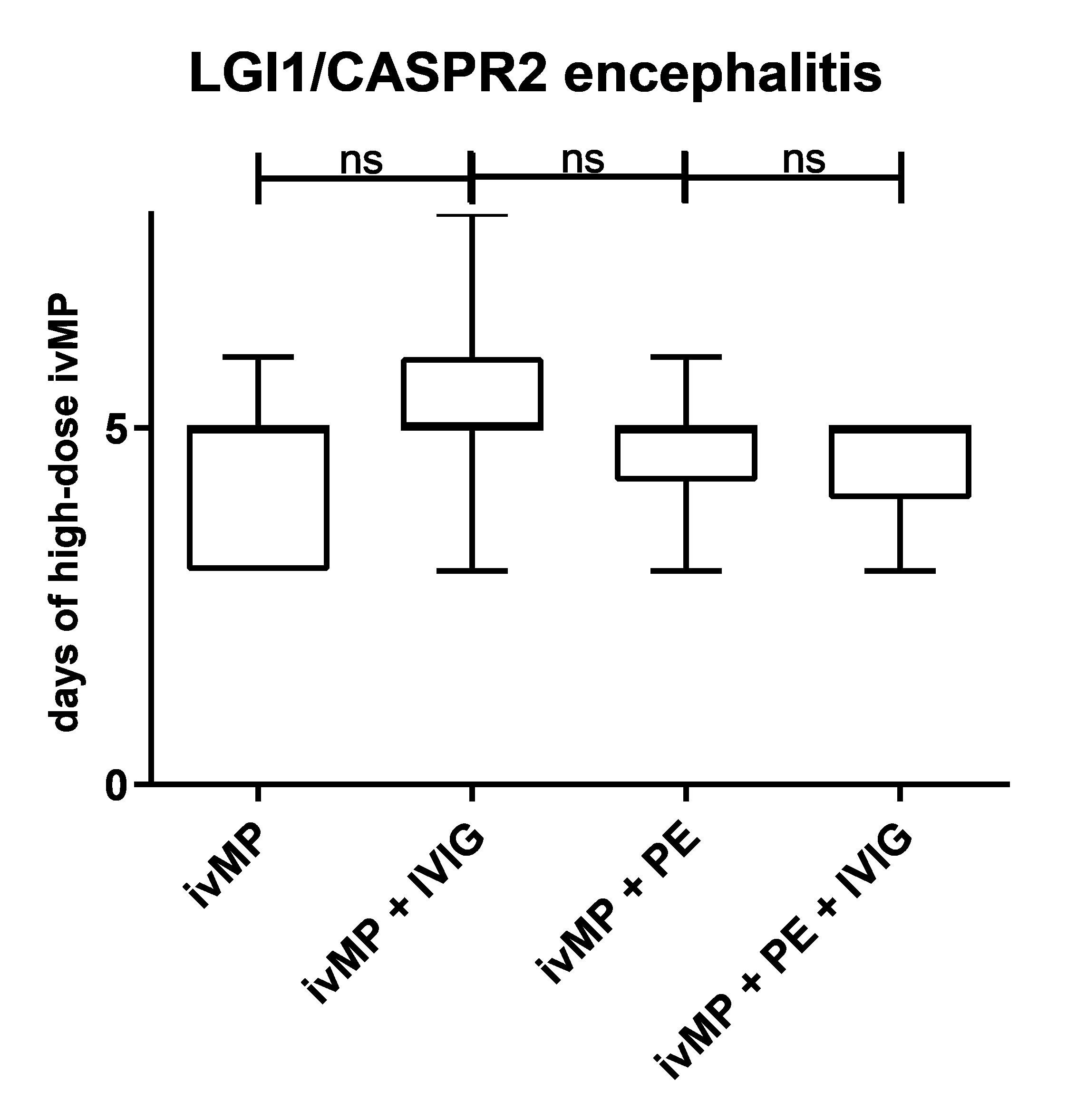

Supplement: Supplementary file 4 — Supplementary file4 (TIF 113 KB) [file 415_2025_13032_MOESM4_ESM.tif]

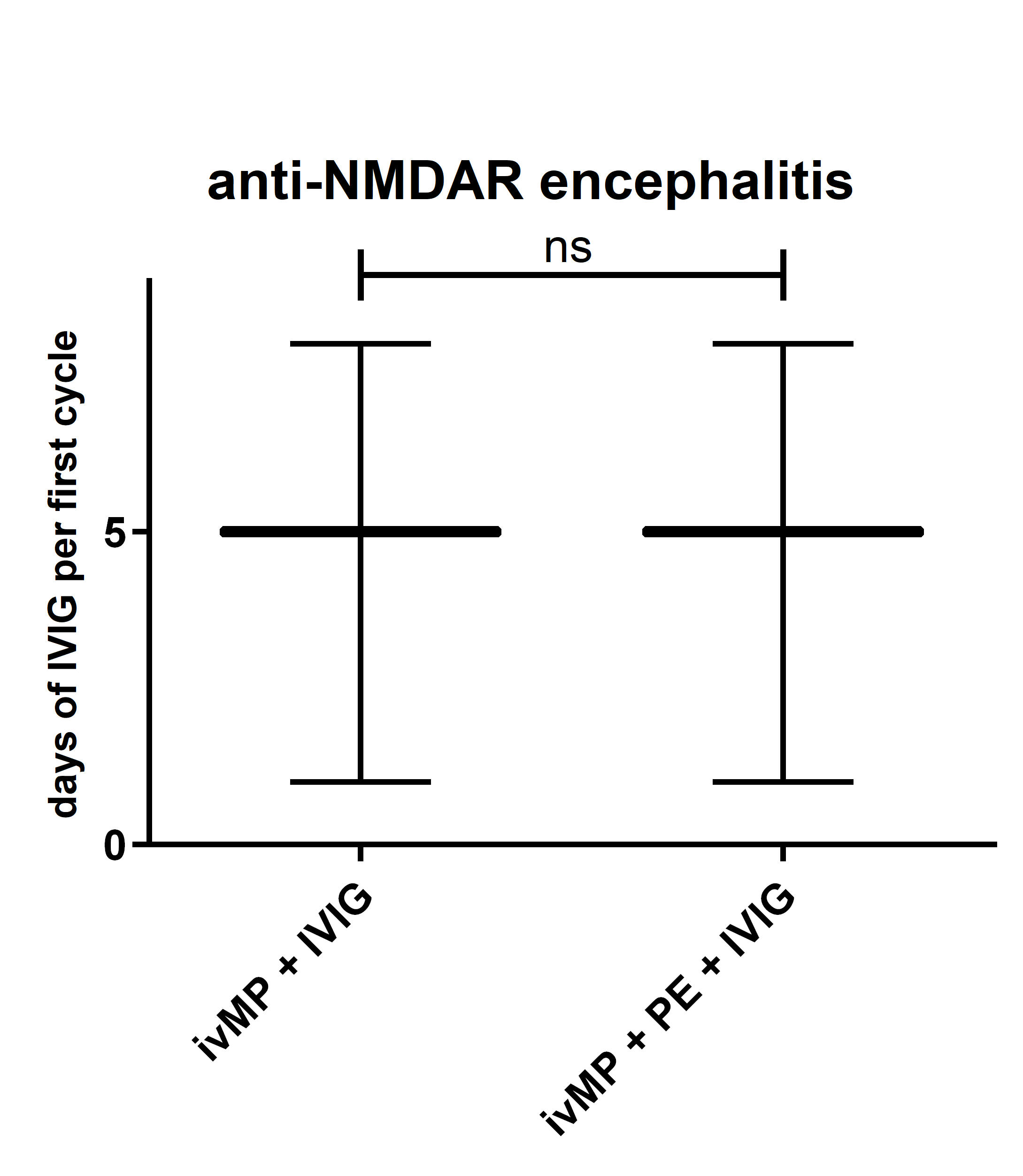

Supplement: Supplementary file 5 — Supplementary file5 (TIF 99 KB) [file 415_2025_13032_MOESM5_ESM.tif]

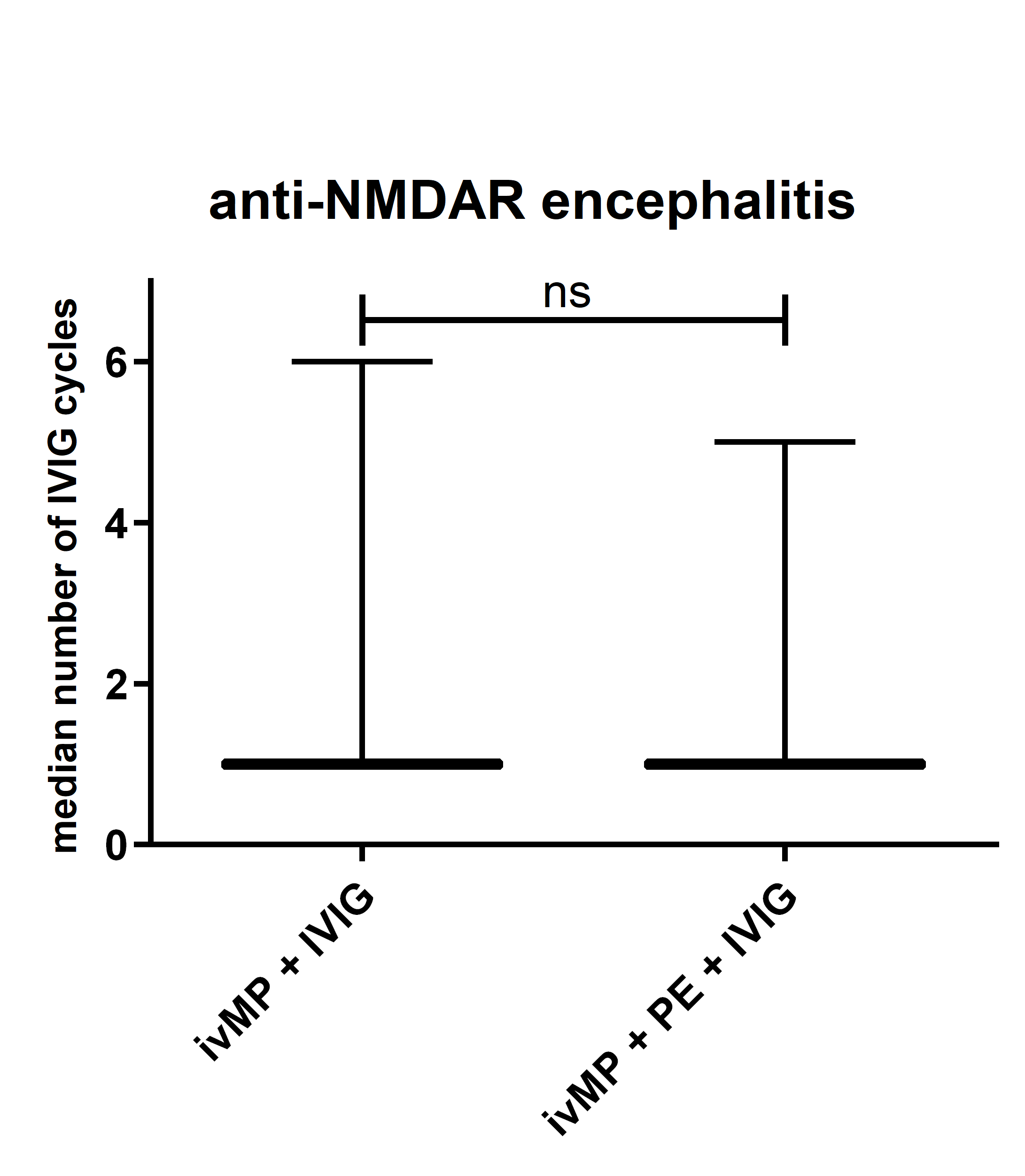

Supplement: Supplementary file 6 — Supplementary file6 (TIF 101 KB) [file 415_2025_13032_MOESM6_ESM.tif]

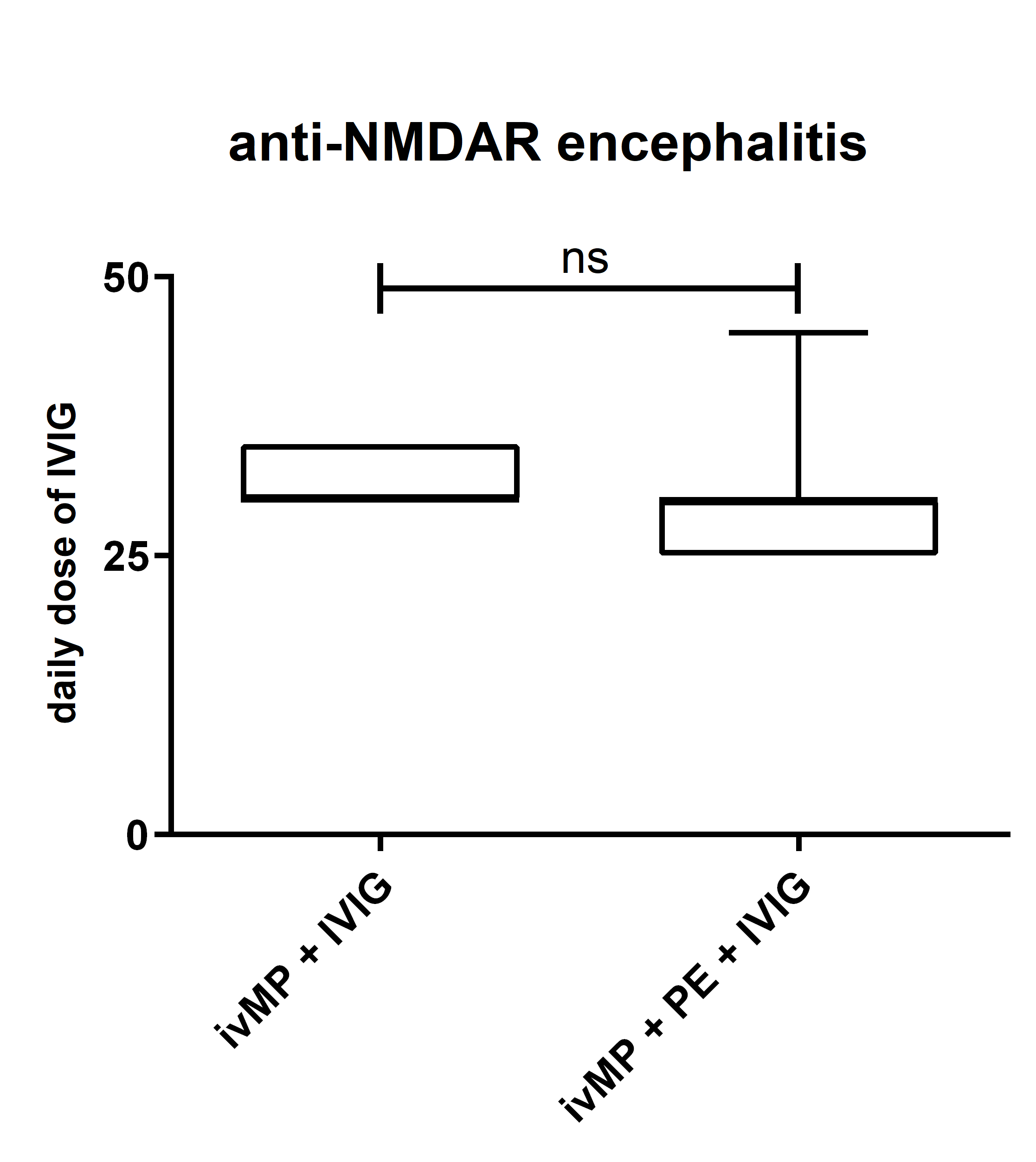

Supplement: Supplementary file 7 — Supplementary file7 (TIF 95 KB) [file 415_2025_13032_MOESM7_ESM.tif]

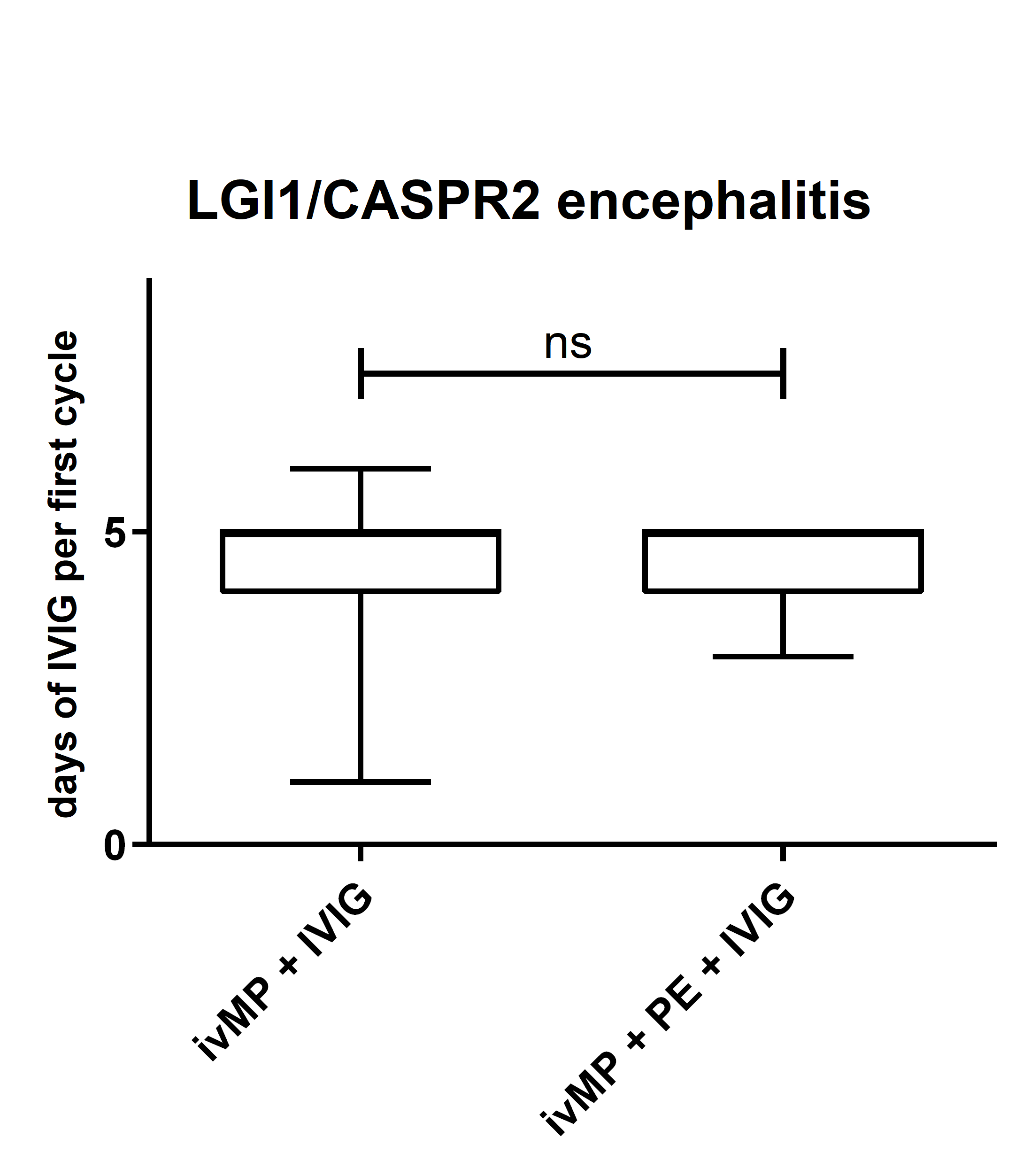

Supplement: Supplementary file 8 — Supplementary file8 (TIF 97 KB) [file 415_2025_13032_MOESM8_ESM.tif]

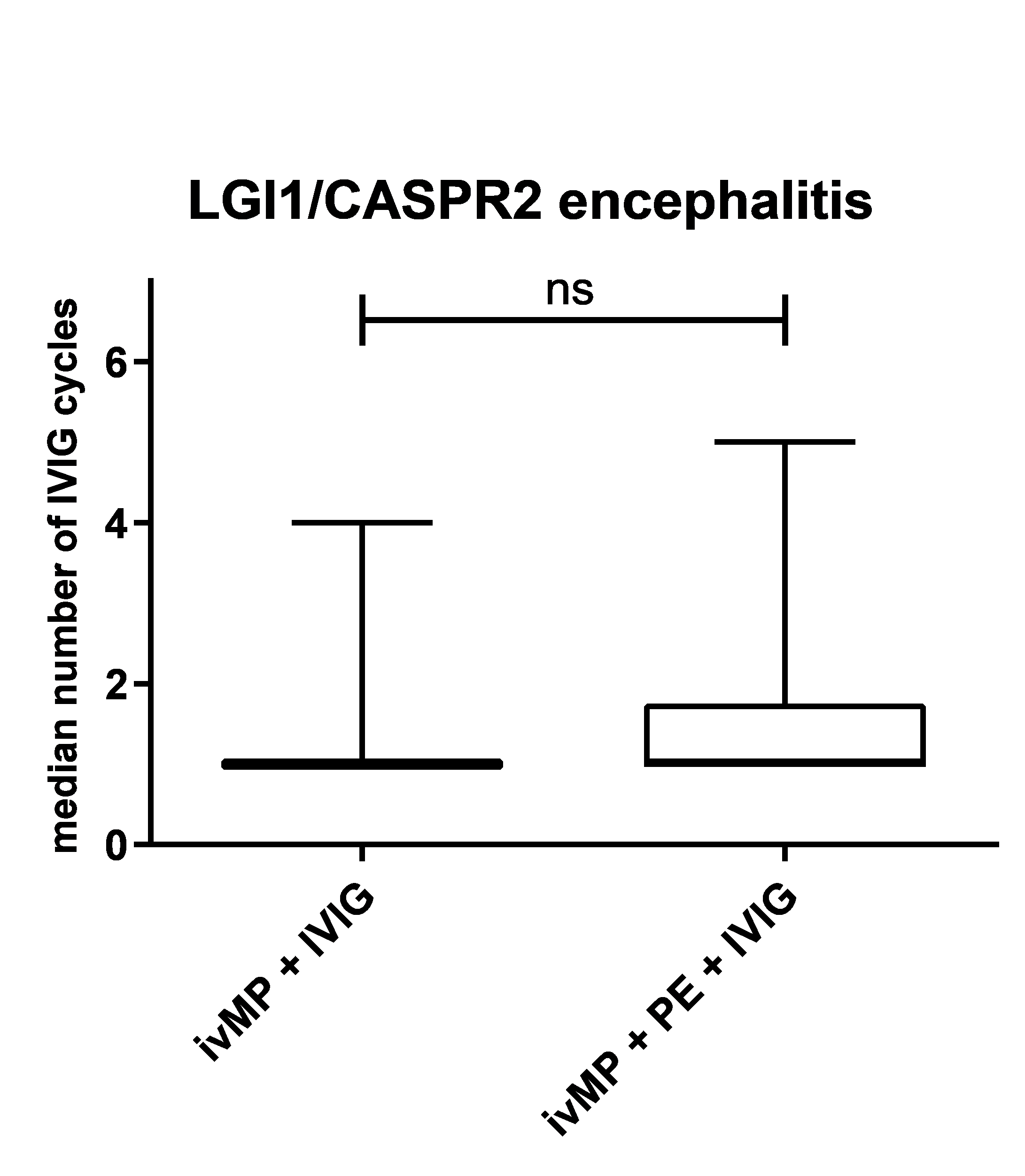

Supplement: Supplementary file 9 — Supplementary file9 (TIF 101 KB) [file 415_2025_13032_MOESM9_ESM.tif]

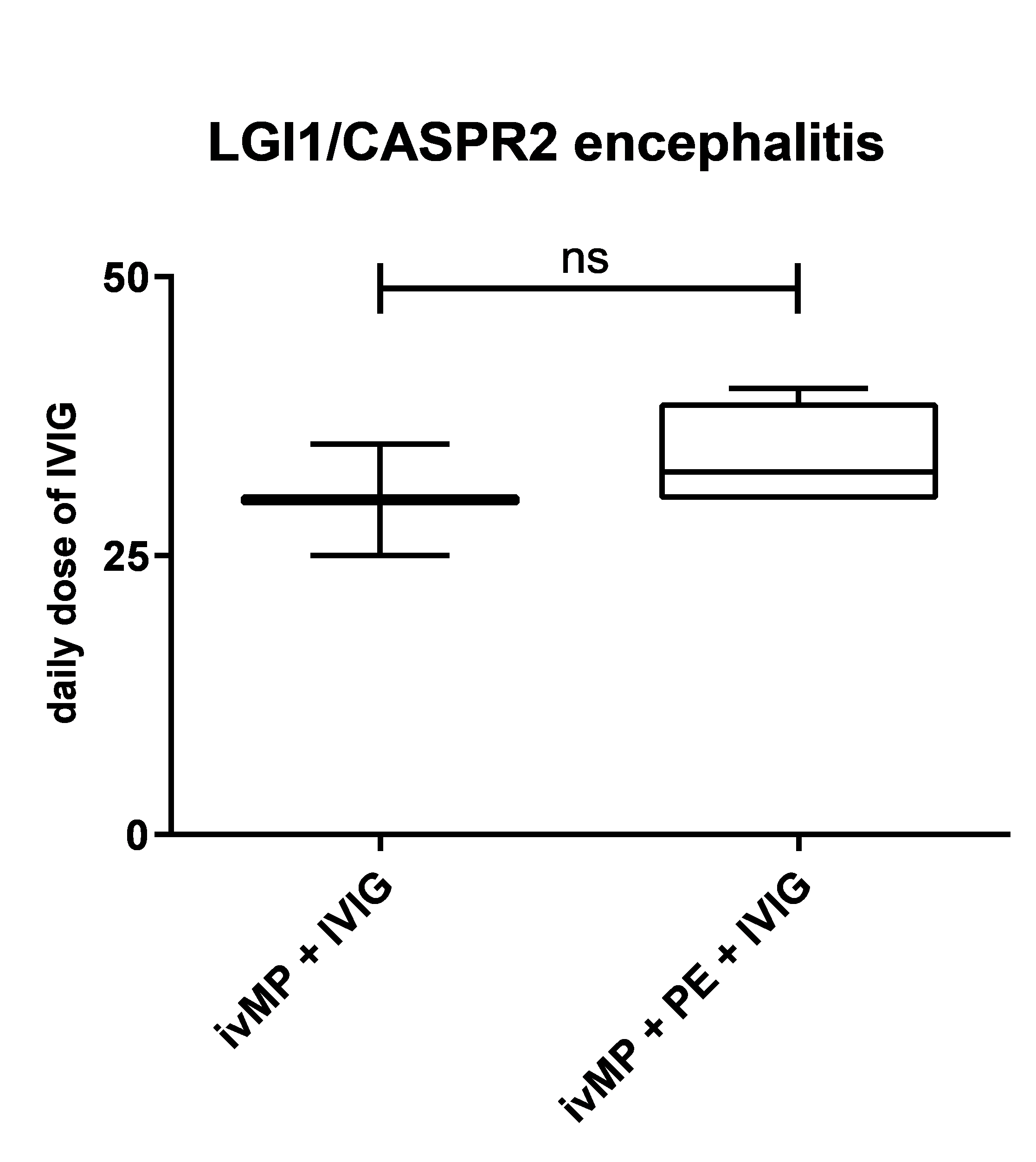

Supplement: Supplementary file 10 — Supplementary file10 (TIF 94 KB) [file 415_2025_13032_MOESM10_ESM.tif]

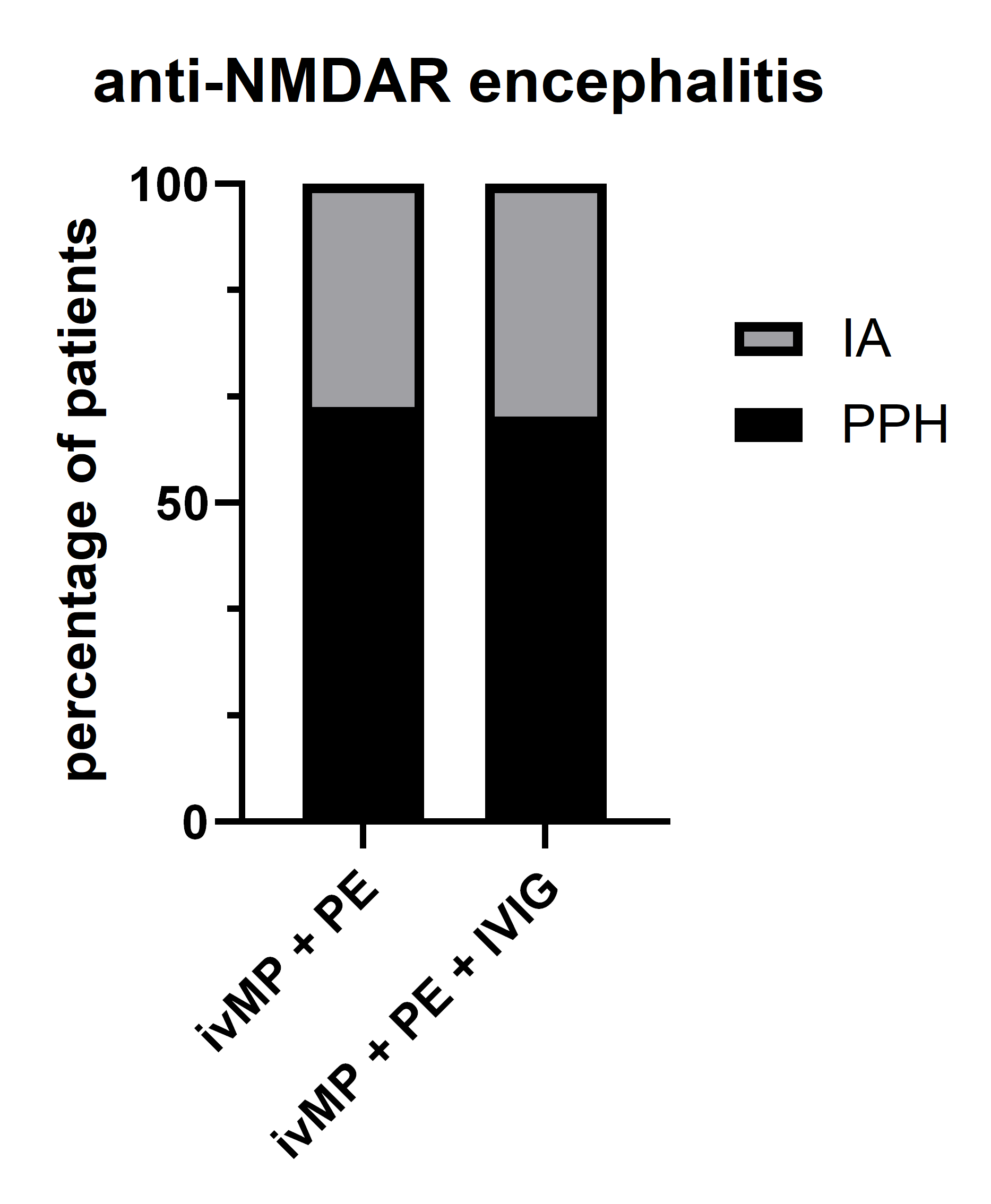

Supplement: Supplementary file 11 — Supplementary file11 (TIF 110 KB) [file 415_2025_13032_MOESM11_ESM.tif]

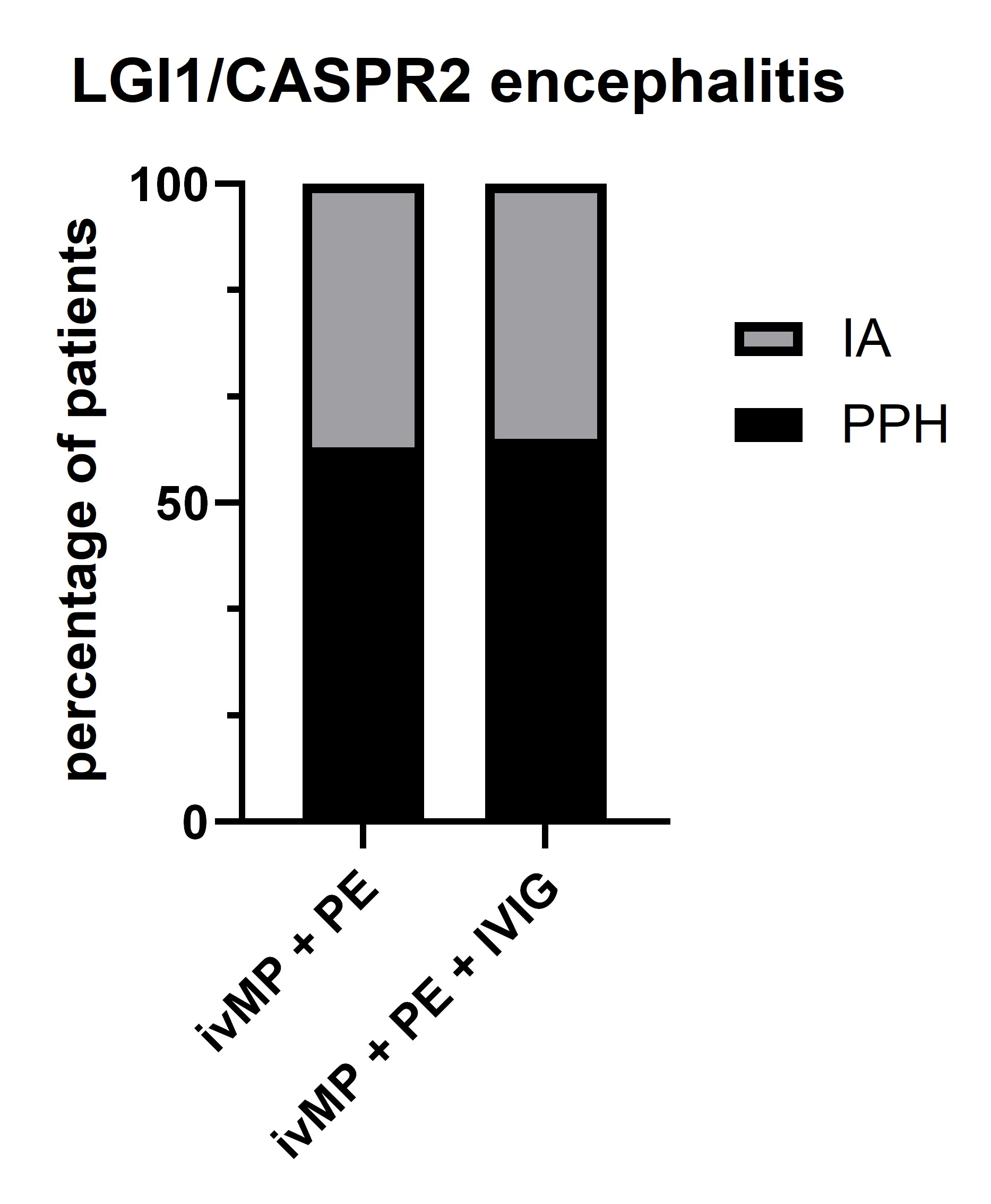

Supplement: Supplementary file 12 — Supplementary file12 (TIF 111 KB) [file 415_2025_13032_MOESM12_ESM.tif]
